# Erianin suppresses constitutive activation of MAPK signaling pathway by inhibition of CRAF and MEK1/2

**DOI:** 10.1038/s41392-023-01329-3

**Published:** 2023-03-06

**Authors:** Penglei Wang, Xuechao Jia, Bingbing Lu, Han Huang, Jialin Liu, Xuejiao Liu, Qiong Wu, Yamei Hu, Pan Li, Huifang Wei, Tingting Liu, Dengyun Zhao, Lingwei Zhang, Xueli Tian, Yanan Jiang, Yan Qiao, Wenna Nie, Xinli Ma, Ruihua Bai, Cong Peng, Zigang Dong, Kangdong Liu

**Affiliations:** 1 Department of Pathophysiology, Basic Medicine Research Center, School of Basic Medical Sciences, AMS, Zhengzhou University, 450000 Zhengzhou, China; 2 China-US (Henan) Hormel Cancer Institute, 450000 Zhengzhou, China; 3 The Department of Pathology, Affiliated Cancer Hospital of Zhengzhou University, 450000 Zhengzhou, China; 4 The Department of Dermatology, Xiangya Hospital, Central South University, 410078 Changsha, China; 5 The Collaborative Innovation Center of Henan Province for Cancer Chemoprevention, 450000 Zhengzhou, China; 6 State Key Laboratory of Esophageal Cancer Prevention and Treatment, Zhengzhou University, 450000 Zhengzhou, China; 7 Provincial Cooperative Innovation Center for Cancer Chemoprevention, Zhengzhou University, Zhengzhou, China

**Keywords:** Target identification, Skin cancer

## Abstract

Constitutive activation of RAS-RAF-MEK-ERK signaling pathway (MAPK pathway) frequently occurs in many cancers harboring RAS or RAF oncogenic mutations. Because of the paradoxical activation induced by a single use of BRAF or MEK inhibitors, dual-target RAF and MEK treatment is thought to be a promising strategy. In this work, we evaluated erianin is a novel inhibitor of CRAF and MEK1/2 kinases, thus suppressing constitutive activation of the MAPK signaling pathway induced by BRAF V600E or RAS mutations. KinaseProfiler enzyme profiling, surface plasmon resonance (SPR), isothermal titration calorimetry (ITC), cellular thermal shift assay, computational docking, and molecular dynamics simulations were utilized to screen and identify erianin binding to CRAF and MEK1/2. Kinase assay, luminescent ADP detection assay, and enzyme kinetics assay were investigated to identify the efficiency of erianin in CRAF and MEK1/2 kinase activity. Notably, erianin suppressed BRAF V600E or RAS mutant melanoma and colorectal cancer cell by inhibiting MEK1/2 and CRAF but not BRAF kinase activity. Moreover, erianin attenuated melanoma and colorectal cancer in vivo. Overall, we provide a promising leading compound for BRAF V600E or RAS mutant melanoma and colorectal cancer through dual targeting of CRAF and MEK1/2.

## Introduction

The RAS-RAF-MEK-ERK signaling transduction cascade (mitogen-activated protein kinase pathway) plays a significant part in regulating cell growth, differentiation, and survival.^1^ However, this pathway is frequently constitutively activated in many tumors harboring RAS or RAF mutations.^2,3^ According to statistics, KRAS mutations occur in 40% of colorectal cancers and 35% of non-small cell lung cancers (NSCLC).^4^ Moreover, nearly 20% of malignant melanomas harbor NRAS mutations.^5^ BRAF V600E mutations occur in 66% of cutaneous melanomas and 25% of colorectal cancers.^6^ Usually, BRAF V600E/K mutations without spatial activation and dimerization of B/CRAF kinases can cause sustained constitutive activation of MAPK signal pathway independent of RAS initiation.

Clinically, inhibitors of RAS per se are still in the clinical trial phase. As an alternative, the combination of RAF and MEK1/2 will be critical for developing practical therapeutic approaches to targeting the MAP kinase pathway. Since the first FDA-approved drug vemurafenib for treating BRAF V600E melanomas, 50–70% of advanced melanoma patients with BRAF V600E mutation had clinically benefited from BRAF inhibitors.^7,8^ However, all these BRAF inhibitors are not effective against cancers without BRAF V600E mutation because of the paradoxical activation of MAPK signaling pathway. Through decades of research, several MEK1/2 inhibitors have been proved effective in combined therapies for BRAF V600E mutation melanoma.^9^ Nevertheless, because of the high gain drug-resistant rate, MEK1/2 inhibitors play an affiliated role in combination with BRAF inhibitors in BRAF V600E mutant melanoma.^10^ Moreover, they are generally less effective in BRAF WT and/or KRAS mutant tumors. In addition, clinical BRAF inhibitor monotherapy resistance also induced resistance to MEK1/2 inhibitors.^11,12^

Coincidentally, accumulating studies show RAF family, to be precise, CRAF isoform, plays a crucial part in oncogenic KRAS-driven cancers.^13–15^ More importantly, elimination of CRAF expression in adult mice does not cause significant toxicities, which provides application prospects for CRAF inhibitors as a suitable therapeutic option.^13^ Intriguingly, pan-RAF inhibitors like LY3009120 were thought effective to overcome the paradoxical activation by inhibiting both B/CRAF monomer and dimmer. However, preliminary clinical data showed the high toxicity limited the effect in tumors. The clinical toxicity of pan-RAF inhibitor LY3009120 may be due to interfering with other RAF isoforms (ARAF or BRAF).^16^ Based on these findings, the inhibition of CRAF but not BRAF can provide an effective therapeutic strategy in KRAS mutant tumors. Therefore, simultaneous targeting CRAF and MEK1/2 is highly anticipated as a strategy to treat KRAS mutant cancers. Moreover, a reverse genetic screen study has demonstrated that reduction of reactivated CRAF induced by MEK1/2 inhibitors enlarges the clinical benefit of the latter in KRAS mutant colorectal cancer (CRC) cell lines.^17^

Naturally sourced, highly effective and low-toxic small molecule compounds are considered as potential therapies for cancer. Erianin, isolated from Chinese medicine Drumstick Dendrobium, has been reported to induce apoptosis, block angiogenesis or inhibit cell growth and migration in multiple cancers through ERK pathway,^18^ ROS-mediated JNK/c-Jun and AKT/mTOR pathways,^19^ PI3K/Akt/mTOR pathway,^20^ and calcium/calmodulin-dependent ferroptosis.^21^ Furthermore, a network pharmacology targets prediction indicated BRAF as a potential target of erianin, in which the MAPK cascade pathway was also enriched.^22^ However, the direct molecular target and correlated signaling pathway for erianin are yet to be identified. In this study, erianin was found as a CRAF and MEK1/2 dual-target inhibitor through the aid of Kinase Profiler™ enzyme profiling assay and its inhibition of disordered MAPK signaling pathway in either BRAF V600E or RAS mutant tumors.

## Results

### Discovery of a direct CRAF and MEK1/2 inhibitor erianin

**Fig. 1:**
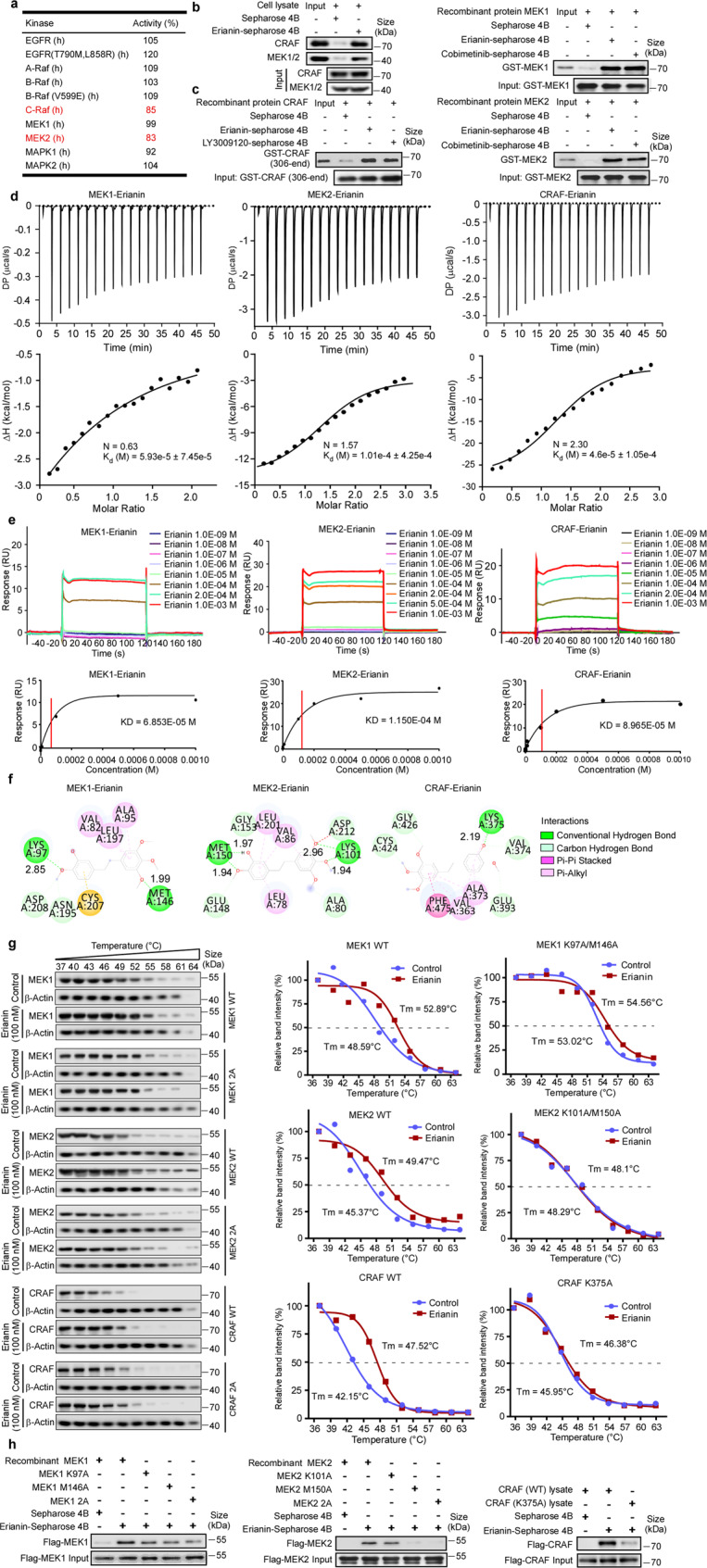
Discovery of a direct CRAF and MEK1/2 inhibitor erianin. **a** KinaseProfiler™ enzyme profiling assay was performed to screen target kinases of erianin. **b** The binding of erianin with CRAF and MEK1/2 present in A375 cell lysate was determined using an erianin-conjugated Sepharose 4B bead pull-down assay. The band was visualized by western blotting. **c** The binding of erianin with human recombinant CRAF (306-end), MEK1 full length and MEK2 full length proteins were evaluated using a erianin-conjugated bead pull-down assay. The band was visualized by western blotting. **d** ITC assay between erianin and MEK1 full length, MEK2 full length or CRAF (306-end). The titration curve was fitted with nonlinear least squares estimation model using Microcal ORIGIN V7.0. **e** SPR was performed using human recombinant CRAF (306-end), MEK1 full length and MEK2 full length proteins with increasing concentrations of erianin. The equilibrium dissociation constant (KD) was evaluated according to the response-concentration curve. **f** Computational docking between erianin and MEK1, MEK2, or CRAF. The figure shows that erianin forms conventional hydrogen bond with MEK1 at both Lys 97 and Met146, MEK2 at both Lys101 and Met150, as well as with CRAF at Lys375. The distance between erianin and protein residues was labeled. **g** Cellular thermal shift assay between erianin and MEK1, MEK2, or CRAF. 293 T cells were transfected with plasmids of pcDNA3.1-3×Flag-C-MAP2K1/MAP2K1 (K97A, M146A), MAP2K2/MAP2K1 (K101A, M150A), or CRAF/CRAF (K375A). The curve was fitted using GraphPad Prism 7.0. **h** The wild-type and mutant MEK1 and MEK2 were expressed in E. coli, the wild-type and mutant CRAF were expressed in 293 T cells. The binding of erianin with recombinant MEK1 and MEK2 proteins or cell lysates was evaluated by an erianin-conjugated bead pull-down assay

To discover multi-kinase inhibitors, a series of natural compounds (the structures as shown in Supplementary Table S1) were screened through phenotypic experiment previously, and erianin exhibited a potent antitumor effect. Following Kinase Profiler™ enzyme profiling assay treated with those compounds, we found erianin inhibited kinase activity of CRAF and MEK2 (Fig. 1a and Supplementary Table S2). To identify the direct protein targets, erianin was conjugated to Sepharose 4B beads and in vitro and ex vivo pull-down assays were conducted. Results showed that erianin directly bound to BRAF, CRAF, and MEK1/2 (Fig. 1b, c and Supplementary Fig. S1a, b) but not other MAPK signaling members, such as EGFR and ERK1/2. To evaluate the binding affinity between erianin and targets (MEK1, MEK2, or CRAF), ITC assay was utilized and according to the titration curve, the affinity (K_d_ values) of MEK1-erianin, MEK2-erianin, CRAF-erianin were 5.93e-5 ± 7.45e-5 M, 1.01e-4 ± 4.25e-4 M, and 4.6e-5 ± 1.05e-4 M, respectively (Fig. 1d). Moreover, these results were also confirmed by using SPR assay, which revealed that erianin had a strong affinity with MEK1, MEK2, and CRAF with KD values 6.85e-05 M, 1.15e-04 M, and 8.97e-05 M, respectively (Fig. 1e). Meanwhile, LY3009120 and cobimetinib were selected as inhibitors of CRAF and MEK1, and the results showed the affinity between erianin and its targets were quite similar to positive control LY3009120-CRAF and cobimetinib-MEK1 (Supplementary Fig. S1c). Collectively, these data indicated that ITC analysis results were consistent with the results of SPR assay. To further explore how erianin interacts with CRAF or MEK1/2, we performed the computational docking model. The docking result demonstrated that erianin formed conventional hydrogen bonds with CRAF Lys 375 (2.19 Å), MEK1 Lys 97 (2.85 Å) and Met146 (1.99 Å), MEK2 Lys101 (1.94 Å and 1.97 Å), and Met150 (1.94 Å and 2.96 Å) (Fig. 1f, Supplementary Fig. S1d). For further confirmation of the docking result, we conducted molecular dynamics simulation to predict the modes of erianin binding with MEK1, MEK2, and CRAF. As shown in bottom panel of Supplementary Fig. S1e, erianin fits well in the hydrophobic pocket generated by residues K97, I99, L115, L118, I126, V127, F129, I141, M146, C207, D208, F209, L215, and M219 of MEK1. Similarly, as shown in Supplementary Fig. S1f, g, erianin also forms extensive hydrophobic and hydrogen bonding interactions with MEK2 or CRAF. All these interactions are conducive to stabilizing the binding complexes. To confirm the computational docking and molecular dynamics simulations results, we performed cellular thermal shift assay after overexpression of targets. The result indicated that erianin could combine with MEK1, MEK2, or CRAF, respectively, and MEK1 K97A/M146A, MEK2 K101A/M150A, or CRAF K375A impaired physical interactions between erianin and MEK1, MEK2, or CRAF (Fig. 1g). Next, we sought to identify the binding sites and implement binding sites mutations in vitro. The result revealed that erianin bound to MEK1 at both Lys 97 and Met146, MEK2 at both Lys101 and Met150, as well as binding to CRAF at Lys375 (Fig. 1h). Taken together, erianin is a potential inhibitor of CRAF and MEK1/2.

### Erianin inhibits constitutive activation of MAPK signaling pathway through suppressing CRAF and MEK1/2 but not BRAF kinase activity

**Fig. 2:**
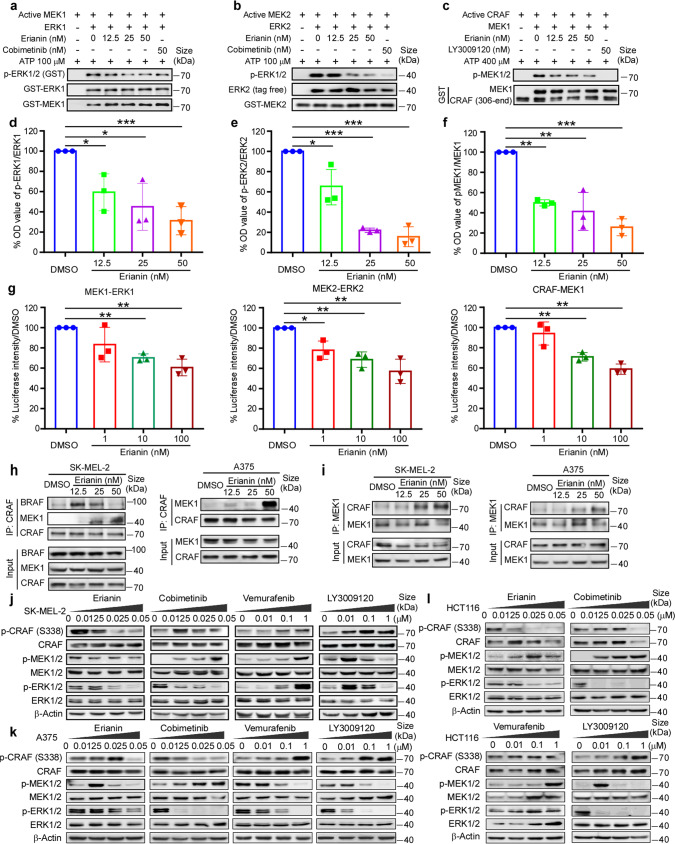
Erianin inhibits MAPK signaling pathway through suppressing CRAF and MEK1/2 but not BRAF kinase activity. **a**, **b** The inhibitory effect of erianin on the activity of MEK1 and MEK2 kinase. Active GST-MEK1 full length or GST-MEK2 full length (60 ng) and various doses of erianin were incubated with inactive GST-ERK1 or tag free ERK2 (400 ng) as substrate at 30 °C for 30 min. The phosphorylation of ERK1/2 (Thr202/Tyr204) was detected by western blotting. **c** The inhibitory effect of erianin on the activity of CRAF kinase. Active CRAF (306-end) (50 ng) and various doses of erianin were incubated with inactive GST-MEK1 (600 ng) as substrate at 30 °C for 30 min. **d**–**f** Quantifications of integrated density in (**a**–**c**) were performed. Data were shown as means ± S.D. of three independent experiments. The asterisks (*p < 0.05, **p < 0.01, ***p < 0.001) indicate a significant difference in the expression of phosphorylation of ERK1 or ERK2 vs total ERK1 or ERK2 in control and erianin-treated group. **g** The luminescent ADP detection assay was developed to detect the luminescence signal of ATP-to-ADP using the same concentration kinases and substrates described in above kinase assay. Three independent repeats were conducted in this experiment. **h** Immunoprecipitation (IP)/WB of endogenous CRAF from lysates of SK-MEL-2 (NRAS mut) and A375 (BRAF V600E) cells treated with DMSO or erianin at 12.5, 25, 50 nM for 24 h. Total lysates were immunoblotted for BRAF, CRAF, and MEK1. **i** IP/WB of endogenous MEK1 from lysates of SK-MEL-2 and A375 cells treated with DMSO or erianin at 12.5, 25, 50 nM for 24 h. Total lysates were immunoblotted for CRAF and MEK1. **j**, **k** Western blotting of phospho-CRAF, phospho-MEK1/2 and phospho-ERK1/2 by erianin, vemurafenib, cobimetinib or LY3009120 at indicated concentration for 24 h in NRAS mutant SK-MEL-2 and BRAF V600E mutant A375 cell lines. **l** Western blotting of MAPK signa**l**ing pathway by erianin, vemurafenib, cobimetinib, or LY3009120 at indicated concentrations for 24 h in KRAS mutant HCT116 cell line

To verify the effect of erianin against CRAF and MEK1/2, we implemented an in vitro kinase assays using active CRAF and MEK1/2 to reveal the phosphorylation of corresponding substrates. The results showed that erianin, at doses ranging from 12.5 to 50 nM, inhibited both MEK1/2 (Fig. 2a, b, d, e) and CRAF (Fig. 2c, f) kinase activity in a dose-dependent manner. However, erianin could not suppress either BRAF WT or BRAF V600E kinase activity using the above doses (Supplementary Fig. S2a, b). To further evaluate the kinase activity alteration after erianin treatment, luminescent ADP detection assay was developed to detect the concentration of ATP to ADP. As illustrated in Fig. 2g (left and middle panel), erianin (1–100 nM) inhibited the kinase activity of MEK1 and MEK2 with a decrease of luminescence signal. Similarly, erianin (1–100 nM) also inhibited the kinase activity of CRAF (Fig. 2g right panel). Enzyme kinetics assay using a series of ATP (1, 5, 10, 50, and 100 μM) indicated erianin inhibited MEK1, MEK2 and CRAF in an ATP dependent manner (Supplementary Fig. S2c). Above all, erianin inhibits both MEK1/2 and CRAF kinase activity. Furthermore, we investigated the downstream RAF-MEK1/2-ERK1/2 signaling. BRAF V600E and RAS mutations are known to constitutively activate MAPK signaling pathway. In RAS-GTP-dependent driven cells, dimerization of B/CRAF plays a major part in activating MAPK pathway. Furthermore, disrupting CRAF-mediated MEK activation is proved essential to effectively inhibit MAPK signaling pathway in KRAS mutant tumors.^17^ Unlike the type I½ BRAF inhibitor vemurafenib, erianin inhibited BRAF-CRAF heterodimer and it was also reflected in the suppression of MAPK signaling pathway in SK-MEL-2 (Fig. 2h, j). Immunoprecipitation results indicated that erianin stabilized CRAF-MEK1 complex, which was coincident with decreased phosphorylated MEK1/2 levels (Fig. 2h–l). Western blotting results showed that erianin inhibited constitutive activation of MAPK signaling pathway in both BRAF V600E and RAS mutant cell lines, without paradoxical activation due to dual target of CRAF and MEK1/2 (Fig. 2j–l). The result also demonstrated that vemurafenib induced the paradoxical activation of MAPK signaling pathway through increasing activation of CRAF, MEK1/2, and ERK1/2 in SK-MEL-2 and HCT116. Even though cobimetinib inhibits phosphorylation of ERK1/2 effectively in both BRAF V600E and RAS mutant cells, actually it induces the increased kinase activity of RAF heterodimer with increased phosphorylation of MEK1/2 in a RAS-dependent manner in SK-MEL-2 and HCT116. Besides, another pan-RAF inhibitor LY3009120 also induced kinase activity of CRAF at a low dose with amplified phosphorylation of CRAF and MEK1/2. Taken together, erianin inhibits constitutive activation of MAPK signaling pathway in both BRAF V600E and RAS mutant cells through suppressing both CRAF and MEK1/2 kinase activity.

### Erianin inhibits proliferation in BRAF V600E or RAS mutant cell lines

**Fig. 3:**
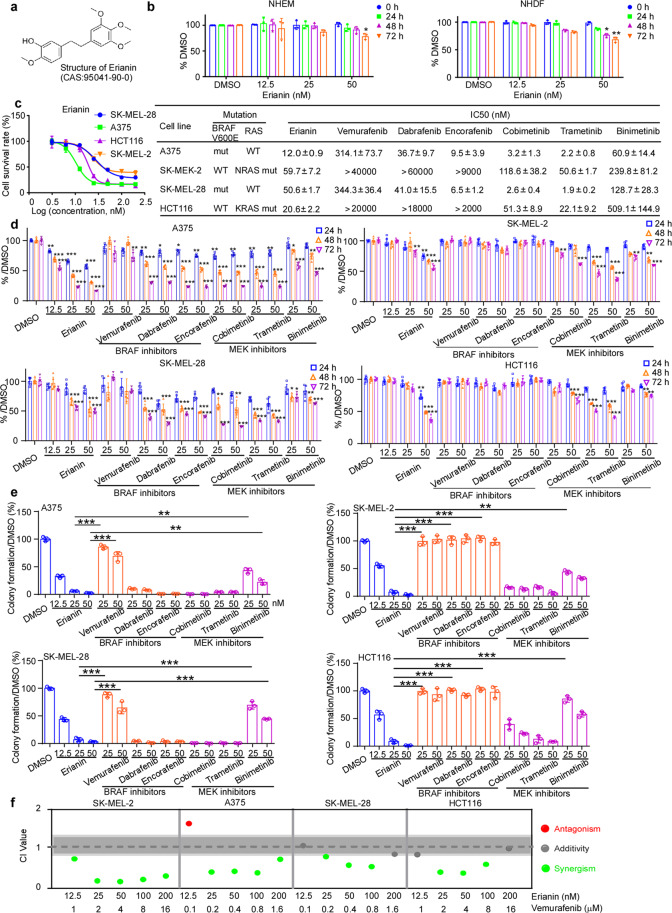
Erianin inhibits proliferation in BRAF V600E or RAS mutant cell lines. **a** Chemical structure of erianin. **b** Cytotoxicity of erianin in normal NHEM and NHDF cell lines using MTT assay. **c** The left panel shows representative dose–response curves by MTT assay. The SK-MEL-2 (NRAS mut), HCT116 (KRAS mut), A375 (BRAF V600E), and SK-MEL-28 (BRAF V600E) cell lines were exposed to erianin for 72 h. The concentrations are transformed to Log10 values; the *Y*-axis shows the corresponding relative cell viability. The right panel shows IC50 values of erianin, three BRAF inhibitors (vemurafenib, dabrafenib and encorafenib) and three MEK inhibitors (cobimetinib, trametinib, and binimetinib) calculated in GraphPad Prism 7.0. **d** The effect of erianin on growth of SK-MEL-2, A375, SK-MEL-28, and HCT 116 cells was estimated by MTT assay at 24, 48, or 72 h. Data were shown as means ± S.D. **e** The effect of erianin on anchorage-independent growth in above cells was evaluated. Data were shown as means ± S.D. Scale bars: 400 μm. The colonies numbers were calculated in Image-Pro Plus software. *p < 0.05; **p < 0.01; ***p < 0.001. **f** Synergism effect of erianin and vemurafenib in SK-MEL-2, A375, SK-MEL-28, and HCT 116. The synergism and antagonism (CI value) were determined and analyzed using CompuSyn 1.0. CI value > 1.1 indicates antagonism, 1.1 ≥ CI value > 0.9 shows addictive effect and CI value ≤ 0.9 indicates synergism

Clinically, union application of BRAF and MEK1/2 inhibitors was the first-line therapy in BRAF V600E mutant advanced melanoma patients. However, the combination therapy exhibited less efficiency in melanoma patients without BRAF V600E mutation partly due to activating unoccupied CRAF protomer. From the TCGA database, mutations of BRAF and RAS mainly exist in melanoma and CRC (Supplementary Fig. S3a–c). BRAF V600E mutant (A375, SK-MEL-28), KRAS mutant (HCT116) and NRAS mutant (SK-MEL-2) cells were selected for anti-proliferation effect using various doses (0, 12.5, 25, 50 nM) of erianin. Those doses were proved to have no toxicity in normal human epidermal melanocytes (NHEM) juvenile and normal human dermal fibroblasts (NHDF) cell lines (Fig. 3b). As a result, erianin acquires a better effect in these cell lines due to dual inhibition of CRAF and MEK1/2, followed by inhibiting the paradoxical activation of MAPK signaling. Besides, we found that IC50 of erianin in A375 (12.0 ± 0.9 nM), SK-MEL-28 (50.6 ± 1.7 nM), SK-MEL-2 (59.7 ± 7.2 nM), and HCT116 (20.6 ± 2.2 nM) (Fig. 3c, Supplementary Fig. S4a) was lower than BRAF V600E inhibitor vemurafenib in cell lines with BRAF mutation and MEK inhibitor cobimetinib in cell lines with RAS mutation. To further define the anti-proliferation of erianin in BRAF or RAS mutant cell lines, we performed the MTT assay and anchorage-independent cell growth assay. Similarly, erianin could markedly suppress proliferation (Fig. 3d) and colonies formation (Fig. 3e, Supplementary Fig. S4b) in both BRAF and RAS mutant cell lines. Furthermore, as a CRAF and MEK1/2 inhibitor, erianin had a synergistic effect with BRAF inhibitor vemurafenib, with most of the combination index (CI) ≤ 1 in both BRAF and RAS mutant cell lines (Fig. 3f).

### Knockdown of CRAF and MEK1/2 can abolish the efficiency of erianin

**Fig. 4:**
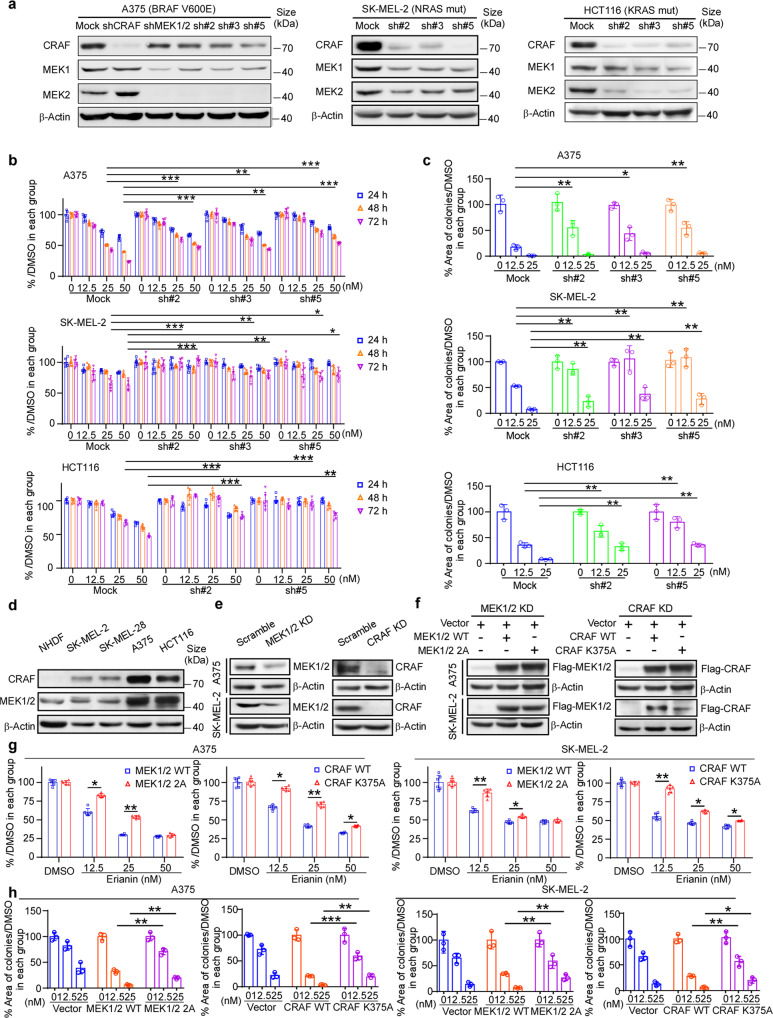
Knockdown of CRAF and MEK1/2 can abolish the efficiency of erianin. **a** The expression of CRAF, MEK1, and MEK2 in A375, SK-MEL-2, and HCT116 cells expressing shRNA-mock or shRNA of both CRAF and MEK1/2 was evaluated by western blotting. **b** MTT assay was evaluated in A375, SK-MEL-2, and HCT116 cells after dual knockdown of CRAF and MEK1/2. The asterisks (*p < 0.05; **p < 0.01; ***p < 0.001) indicate a significant difference between shRNA-mock and shRNA-CRAF/MEK1/2 cells. **c** Anchorage-independent growth was assessed in A375, SK-MEL-2, and HCT116 cells expressing shRNA-mock or shRNA-CRAF/MEK1/2. Positive colony areas in anchorage-independent growth were calculated using Image-Pro Plus software. Scale bars: 400 μm. Data were shown as means ± S.D. The asterisks (*p < 0.05; **p < 0.01) indicate a significant difference. **d** Expression of CRAF and MEK1/2 in melanoma and colorectal cancer cell lines. **e** Western blotting of CRAF or MEK1/2 after knocking down CRAF or MEK1/2 in A375 and SK-MEL-2 cell lines. **f** Western blotting of Flag-CRAF or Flag-MEK1/2 after transfected with mutant CRAF or MEK1/2 in A375 and SK-MEL-2 cell lines described in (**e**). **g** Rescue assay (MTT) after knocking down MEK1/2 or CRAF followed by overexpression of MEK1/2 WT/2 A or CRAF WT/K375A, respectively, in A375 and SK-MEL-2 cell lines. **h** Rescue assay (anchorage-independent growth assay) after knocking down MEK1/2 or CRAF followed by overexpression of MEK1/2 WT/2 A or CRAF WT/K375A respectively in A375 and SK-MEL-2 cell lines. Data were shown as means ± S.D. Scale bars: 400 μm. The asterisks (*p < 0.05; **p < 0.01) indicate a significant difference

The results above reveal that erianin can effectively inhibit proliferation in cell lines harboring aberrant activation of RAF-MAPK signaling pathway. To more closely explore the essential of targets CRAF and MEK1/2 to erianin, we tested efficiency of erianin after knockdown of CRAF and MEK1/2 by transfecting shRNA into cancer cell lines (Fig. 4a). As a result, after CRAF and MEK1/2 were both knocked down, the cell viability of A375 with erianin treatment in these CRAF and MEK1/2 knockdown groups was higher than that in the mock group (Fig. 4b top-panel). Similarly, knockdown of CRAF and MEK1/2 could also repeal sensitivity of erainin in SK-MEL-2 (Fig. 4b middle panel) and HCT116 (Fig. 4b bottom panel) compared with the mock group. Moreover, the inhibitory effect of erianin in colony formation was also impaired after CRAF and MEK1/2 knockdown (Fig. 4c and Supplementary Fig. S5a). Systemic elimination of MEK1/2 alleles in adult mice is deadly for severe intestinal defects, and CRAF was reported indispensable in RAS-driven tumors.^13,23^ Here, depletion of MEK1/2 or CRAF was performed by using CRISPR/Cas9 in A375 and SK-MEL-2 cell lines to verify erianin’s inhibitory effect. Either MEK1/2 or CRAF knockout impaired the effect of erianin significantly (Supplementary Fig. S5c–f). To further confirm whether CRAF or MEK1/2 was required for anti-proliferation effect of erianin, CRAF or MEK1/2 was knocked down in A375 and SK-MEL-2, then the mutant CRAF or MEK1/2 was transfected into cancer cell and rescue assay was performed (Fig. 4d–h). Either CRAF or MEK1/2 mutations reduced the efficacy (MTT and anchorage-independent cell growth) of erianin (Fig. 4g, h and Supplementary Fig. S5b). These results indicate that either CRAF or MEK1/2 plays a vital part in erianin inhibiting cell proliferation.

### Erianin suppresses either BRAF V600E or RAS mutant cell growth in CDX model through targeting CRAF and MEK1/2

**Fig. 5:**
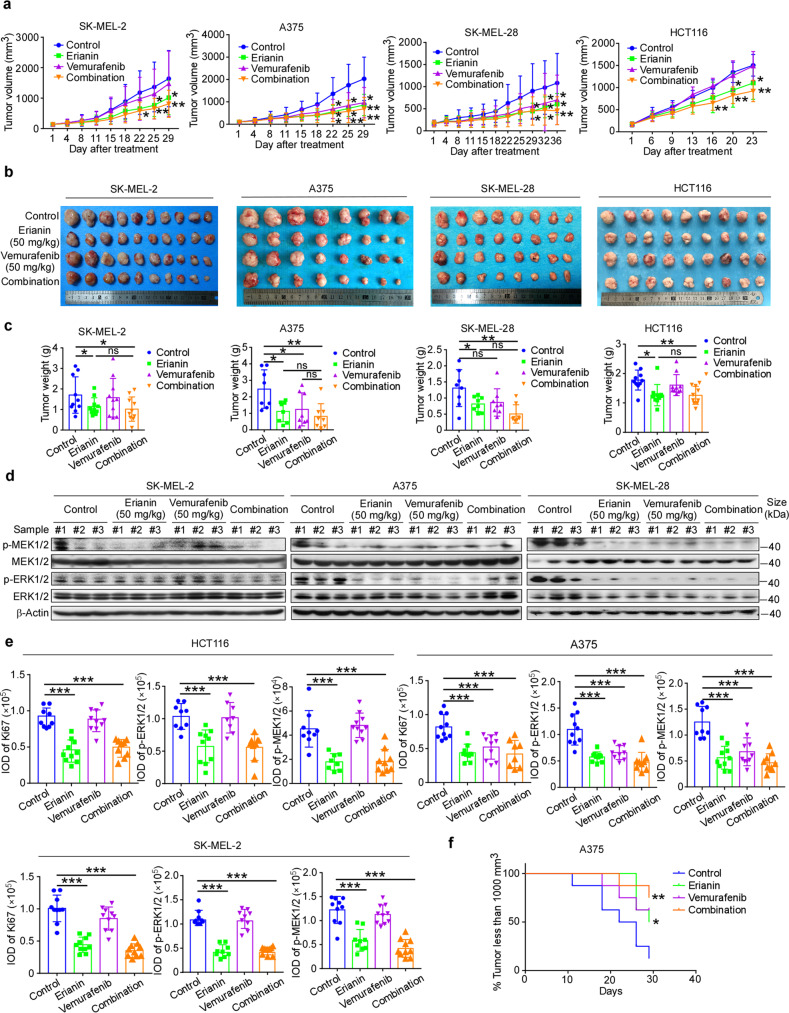
Erianin suppresses either BRAF V600E or RAS mutant cell growth in CDX model. **a** NOD-SCID mice were injected subcutaneously with SK-MEL-2 (NRAS mut, 5 × 10^6^ cell/mouse), A375 (BRAF V600E, 1 × 10^7^ cell/mouse), SK-MEL-28 (BRAF V600E, 5 × 10^6^ cell/mouse) and HCT116 (KRAS mut, 1 × 10^7^ cell/mouse) cells mixed with Matrigel (1:1); erianin (50 mg/kg), vemurafenib (50 mg/kg) or the combine was given through oral gavage and the size of the tumors was monitored twice per week. Tumor volume (mm^3^) = (length × width × height) × 0.52. SK-MEL-2: *n* = 10; A375 and SK-MEL-28: *n* = 8; HCT116: *n* = 9. **b** The photographs show tumors from CDX mice treated with vehicle, erianin, vemurafenib, or the combination. **c** The weight of the tumors was quantified and expressed as the treatment groups compared with the vehicle-treated group. Data were presented as mean ± S.D. One-way ANOVA test. *p < 0.05; **p < 0.01. **d** Western blotting shows the expression of phospho-MEK1/2 and phospho-ERK1/2 by erianin in SK-MEL-2, A375, and SK-MEL-28 CDX tumor tissues. The tissue lysates were prepared from CDX tumor tissues in each treatment group. Three samples were randomly prepared for each group and every blot shows one sample. **e** The quantization (IOD values) of IHC staining in the treatment groups compared with the vehicle-treated group. Each point represents the IOD values of four quantified data from one mouse. Scale bars: 50 μm. One-way ANOVA test. ***p < 0.001. **f** Kaplan–Meier curve depicting tumors less than 1000 mm^3^ in the treatment groups compared with the vehicle-treated group

**Fig. 6:**
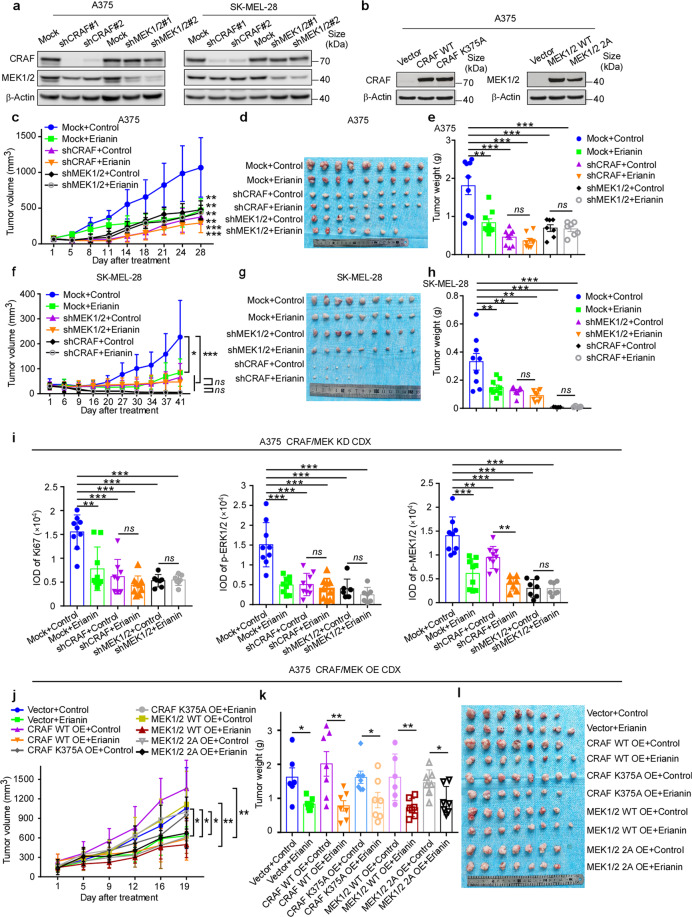
Erianin inhibits CDX tumor growth by targeting CRAF and MEK1/2. **a** Expression of CRAF, MEK1/2 in A375 and SK-MEL-28 after transfecting with shRNA-mock or shRNA of CRAF or MEK1/2 by western blotting. **b** Expression of CRAF, MEK1/2 in A375 after transfecting with pCDH-PURO-3×Flag-CRAF WT/K375A or MEK1/2 WT/2 A. **c**, **f** Tumor volume curve over time in A375 and SK-MEL-28 CDX, TV (mm^3^) = (length × width × height) × 0.52. **d**, **g** The photographs show tumors in A375 and SK-MEL-28 CDX. **e**, **h** The weight of the tumors was quantified between groups in A375 and SK-MEL-28 CDX. Data were presented as mean ± S.D. One-way ANOVA test. *p < 0.05; **p < 0.01. **i** The quantization (IOD values) of IHC staining in the treatment groups compared with the vehicle-treated group. Each point represents the IOD values of four quantified data from one mouse. Scale bars: 50 μm. One-way ANOVA test. ***p < 0.001. **j** Tumor volume curve over times in A375 CRAF WT/K375A or MEK1/2 WT/2 A overexpression CDX model. **k** The weight of the tumors of (**j**). **l** The photographs show tumors of (**j**)

Cell-derived xenograft melanoma and colorectal cancer were utilized to extend the antitumor activity of erianin in vivo. Results showed that in comparison with the vehicle-treated group in A375 and SK-MEL-28, there was a significant decrease in tumor volume in the group of erianin/vemurafenib monotherapy or combination at 50 mg/kg (*p* < 0.05, *p* < 0.05, *p* < 0.01, respectively) (Fig. 5a–c), and there was no significant reduction in body weight (Supplementary Fig. S6a). Consistent with phenotypes observed in vitro, erianin inhibited tumor volume and tumor weight significantly (*p* < 0.05), whereas vemurefinib had a poor effect in SK-MEL-2 and HCT116 CDX model. In vitro study, erianin was observed to have a synergistic effect with BRAF inhibitor vemurafenib, with most of the combination index (CI) ≤ 1. Although combination therapy group did not substantially decrease tumor volume when compared to the erianin monotherapy, it is noteworthy that there was a significant difference in tumor volume between the combination group and the vehicle group (*p* < 0.01). In addition, western blotting showed that erianin and combination group but not vemurafenib significantly decreased phosphorylation of ERK1/2 in SK-MEL-2 CDX tissues; erianin, vemurafenib and the dual-therapy group all significantly decreased phosphorylation of ERK1/2 in BRAF mutant CDX tissues (Fig. 5d, Supplementary Fig. S6b). Furthermore, IHC staining showed that Ki-67, p-ERK1/2 and p-MEK1/2 in the erianin and combination therapy group significantly decreased compared with the vehicle group in SK-MEL-2, A375, and HCT116 cancer tissues (Fig. 5e, Supplementary Fig. S6c). Although Ki-67, p-MEK1/2, and p-ERK1/2 protein levels in vemurafenib-only group decreased compared with the placebo group in A375 cancer tissues, it failed to block the activation of RAS-MAPK signaling in BRAF wild-type CDX tissues. Notably, there was a significant difference in tumor progress between the combination therapy group and the vehicle group in A375 CDX model (*p* < 0.01) (Fig. 5f). Collectively, these data indicate that the antitumor efficacy of erianin in melanoma and colorectal cancer is mediated by reducing MAPK signaling pathway. To test the necessity of CRAF or MEK1/2 involved in the antitumor effect of erianin, knocking down CRAF or MEK1/2 was developed in A375 and SK-MEL-28 CDX, and the result indicated that either CRAF or MEK1/2 deficiency significantly inhibited tumor growth in A375 and SK-MEL-28, as well as reducing the efficiency of erianin in vivo (Fig. 6c–h). Either CRAF (MEK1/2) knockdown or erianin inhibits MAPK signaling pathway (Fig. 6i, Supplementary Fig. S6d). Furthermore, we also verified the effect of erianin after overexpression of CRAF WT/K375A or MEK1/2 WT/2 A in vivo. The growth rate of tumor with high expression of CRAF WT/K375A or MEK1/2 WT/2 A increased and these cells with high expression of wild-type CRAF or MEK1/2 were more sensitive to erianin compared with vector and CRAF K375A or MEK1/2 2 A groups (Fig. 6j–l). In sum, we confirmed erianin suppresses cell-derived xenograft by inhibition of CRAF and MEK1/2.

### Erianin exerts antitumor efficacy in melanoma and colorectal cancer in vivo

**Fig. 7:**
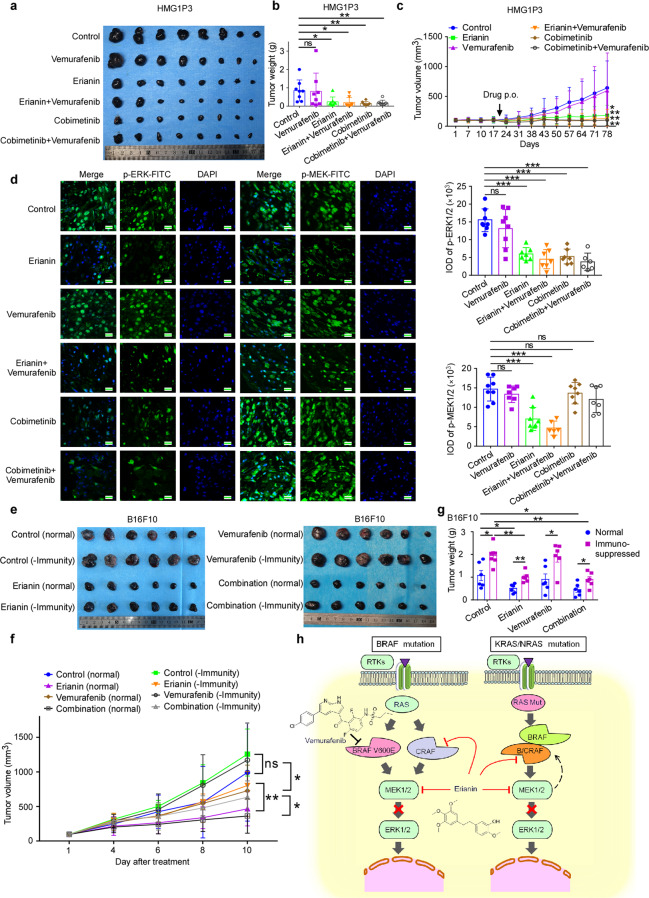
Erianin exerts antitumor efficacy in melanoma and colorectal cancer in vivo. **a** Tumor pharmacodynamic assay was performed in tumor-bearing NPG mice (tumor has been passaged from melanoma patient to mice for three generations). The photographs show tumors from melanoma PDX mice treated with vehicle or drugs. **b** The effect of erianin on the volume of PDX tumors over time (within 78 days) was plotted. Vehicle, erianin (50 mg/kg, once a day), vemurafenib (50 mg/kg, once a day), erianin and vemurafenib combination therapy, cobimetinib (5 mg/kg, twice a week) or vemurafenib and cobimetinib combination therapy (once a day and twice a week, respectively) were administered by oral gavage, *n* = 8 in each group. Tumor volume was measured once a week. One-way ANOVA test. *p < 0.05; **p < 0.01. **c** Tumor weight was measured after treatment on the last day of the study. **d** The expression of phospho-MEK1/2 and phospho-ERK1/2 were examined by immunofluorescence analysis. Scale bars: 20 μm. One-way ANOVA test. ***p < 0.001. **e** Antitumor efficacy of erianin with or without immunity using B16F10 cell xenograft in C57BL-6J mouse. **f**, **g** Trend of tumor volume over time and tumor weight was measured after treatment on the last day of the study. One-way ANOVA test. *p < 0.05; **p < 0.01. **h** The model depicts that erianin suppresses constitutive activation of MAPK signaling pathway in either BRAF V600E or RAS mutant cancers (Created with BioRender.com). Through inhibition of CRAF and MEK1/2 kinases, erianin suppresses phospho-MEK1/2 and phospho-ERK1/2 without paradoxical activation in vitro and in vivo

Based on the mutation of N/KRAS, two PDX cases were selected, in which the expression of CRAF and MEK1/2 were high in tumors (Supplementary Fig. S7a, b). As illustrated in Fig. 7a–c and Supplementary Fig. S7h, compared to the vehicle-treated group, there was a significant decrease in tumor volume in melanoma PDX in the group of erianin and erianin/vemurafenib dual-therapy group, without significant changes in body weight. Nevertheless, vemurafenib monotherapy did not suppress tumor volume significantly. We also used cobimetinib and cobimetinib/vemurafenib combination as positive control and there were no differences between erianin and cobimetinib, as well as between erianin/vemurafenib combination and cobimetinib/vemurafenib combination group. Decrease of phospho-MEK1/2 was only observed in erianin and erianin/vemurafenib dual-therapy group, and even all erianin, erianin/vemurafenib dual-therapy, cobimetinib, and cobimetinib/vemurafenib dual-therapy group inhibited phosphorylation of ERK1/2 (Fig. 7d). Such results were also verified in colorectal cancer harboring KRAS mutation that erianin restrained tumor growth significantly and induced low toxicity in body weight (Supplementary Fig. S7c–f). HE staining was evaluated to identify neither erianin group nor erianin-vemurafenib combination therapy showed toxicity sign through the entire studies (Supplementary Fig. S7f–i). Several clinical studies reveal that immune checkpoint therapies PD-1/PD-L1 synergistically promote RAF inhibitor in treatment of melanoma. Therefore, we checked the immune response involved in antitumor effect of erianin in vivo. After immunosuppression by hydrocortisone (50 mg/kg) for 1 week, B16F10 cell was implanted into C57BL-6J mice followed by vehicle or drug treatment. As revealed in Fig. 7e–g, erianin with innate immunity inhibited tumor volume and tumor weight significantly (*p* < 0.01) compared with the erianin-immunosuppressive group. Supporting this finding, we noticed that CD8a^+^, CD4^+^ T cells, and NK cells increased significantly after treated with erianin (Supplementary Fig. S8e). Moreover, erianin promoted immune cell expression in melanoma PDX without directly inhibiting PD-L1 expression (Supplementary Fig. S8b, d, f). In sum, erianin inhibits melanoma and colorectal cancer in vivo. In view of erianin’s promotion of immune response and upregulation of PD-L1 expression levels, which is a cornerstone for combination strategies, we performed combination therapy of erianin and PD-L1 antibody in B16F10 CDX. Even though erianin or PD-L1 monotherapy inhibited tumor growth, we did not observe significant synergistic effect (Supplementary Fig. S8i, j). Moreover, consistent with Supplementary Fig. S8e, erianin and combination therapy enhanced the immune response by stimulating expression of CD4^+^ and CD8a^+^ cells in spleen, compared with PD-L1 single therapy or the control group (Supplementary Fig. S8k, l). Considering feasibility of erianin as a potential drug candidate, we also performed pharmacokinetics assay. As shown in Supplementary Fig. 9b, erianin has a poor gastrointestinal absorption or high first-pass metabolism in liver for the oral bioavailability was estimated to be 8.0 ± 1.1%.

## Discussion

For decades, although patients harboring BRAF V600E mutation have derived clinical benefit from BRAF inhibitors including vemurafenib, dabrafenib, and encorafenib, acquired resistance and short median progression-free survival (less than 6 months) bring to light the combined therapy with MEK inhibitors.^10,24–26^ Interestingly, MEK inhibitor monotherapy reversely activates RAF kinase activity RAS-dependently and combined treatment with RAF inhibitor can promote the efficiency of MEK inhibitor.^2,27^ According to the previous reports, we hypothesize that inhibition of both CRAF and MEK1/2 is a promising therapeutic strategy for BRAF or RAS-driven tumors. In this paper, we utilized KinaseProfiler™ enzyme profiling assay to exhibit that erianin can be a potential CRAF and MEK inhibitor (Fig. 1a, Supplementary Table S2). In addition, pull-down assay, ITC, SPR assay were performed to confirm this binding effect (Fig. 1b–e, Supplementary Fig. S1a–c). Structure study of ATP-bound MEK–trametinib indicates trametinib inhibits the enzyme through combining with a series of amino acids including Lys97/101, Asp208/212, Ser218/216, and Met143/147 in MEK1/2.^28^ In this study, we observed Lys97/101 and Met146/150 in MEK1/2 and Lys375 residue in CRAF were required for the binding and efficacy of erianin.

Although erianin bound to BRAF, CRAF, and MEK1/2 in vitro and ex vivo binding assay, it inhibited CRAF and MEK1/2 but not BRAF kinase activity (Fig. 2a–g, Supplementary Fig. S2a). Several studies indicated erianin impacted ERK and AKT/mTOR pathways,^18–20^ and the cross-talk between the RAF-MAPK and AKT/mTOR pathways in the proliferation was noticed through the interaction of CRAF and AKT.^29^ Mechanism analysis indicated that CRAF protomer activation and enhancement of phospho-AKT partly revealed the resistant mechanism of BRAF inhibitors.^30^ Usually, BRAF inhibitors induce paradoxical activation of MAPK pathway by reinforcing dimerization of BRAF-CRAF in RAS-driven cancers. Interestingly, studies show that BRAF can activate CRAF, but not the other way around.^31,32^ Combined inhibition of MEK1/2 and CRAF but not BRAF provides a reliable therapeutic strategy in KRAS mutant tumors.^16^ Consistent with published reports, erianin inhibited proliferation of both N/KRAS mutant and BRAF V600E mutant cell lines, whereas BRAF inhibitors only work in BRAF V600E mutant cell lines and MEK inhibitors showed a poor effect in both SK-MEL-2 and HCT116 cells (Fig. 3c–e). Furthermore, we also found BRAF inhibitor or MEK inhibitor monotherapy initiated negative feedback loops in RAS mutant cancer cells, leading to dysregulation of MAPK signaling pathway.

Constitutive activation of MAPK pathway frequently by RAS or BRAF oncogenic mutations show huge potential for B/CRAF and MEK1/2 target therapy. However, paradoxical activation of single BRAF or MEK1/2 inhibitor has always plagued the application in BRAF WT and RAS mutant patients. As expected, erianin inhibited constitutive activation of MAPK signaling pathway in both BRAF and RAS-driven cell lines, without paradoxical activation due to its unique properties (Fig. 2j–l). In RAS-GTP-dependent driven cells, dimerization of BRAF and CRAF is required for robust kinase activity and activation of MAPK pathway. Erianin targets CRAF isoform, decreasing the formation of BRAF-CRAF heterodimers in SK-MEL-2 cell line (Fig. 2h), which was different from pan-RAF inhibitor LY3009120 and MEK inhibitor cobimetinib.^2,30^ Although LY3009120 and cobimetinib both elevate BRAF-CRAF heterodimers, their mechanisms are different. LY3009120 suppresses BRAF-CRAF heterodimer activity and downstream signaling despite it strengthening formation of BRAF-CRAF heterodimers; by contrast, cobimetinib promotes kinase activity of RAF heterodimer in KRAS-dependent tumors by increasing the BRAF-CRAF heterodimers.

We observed erianin inhibited phosphorylation of CRAF S338 in SK-MEL-2 (Fig. 2j). Although LY3009120 (1 μM) alone could inhibit the MAPK pathway, because of CRAF S338 autophosphorylation induced by LY3009120, it shows promotion of downstream MEK1/2 and ERK1/2 at low concentration and inhibition at high concentration. Besides RAS-GTP, CRAF S338 phosphorylation is also motivated through activating MEK1/2, and MEK inhibitors inversely suppress the phosphorylation.^31^ Erianin exhibits similar synergistic activity through inhibiting CRAF and MEK1/2, which has great potential in RAS mutant tumors.

In terms of clinical application for KRAS mutant cancer, MEK inhibitors are observed to have little efficacy clinically, and MEK inhibitors for RAS mutant tumors per se have not yet been accepted into clinical trials. Erianin shows a better effect than vemurafenib in both KRAS mutant and BRAF V600E mutant CDX, which is consistent with our prior phenotype and indicates its potential for target therapy. Additionally, knocking down and overexpression of targets in vivo confirm that erianin inhibits the tumor growth exactly through CRAF and MEK1/2. We observed erianin and BRAF inhibitor had potential synergistic effects at most paired concentration combinations in vitro (Fig. 3f). Although we did not see such effects of erianin and vemurafenib in all CDX mice model, an additivity effect was still observed and higher tolerance was identified in the mice treated with the combination, which led to minimal toxicity after treatment (Fig. 5a–c). Furthermore, our results demonstrated erianin monotherapy already strongly suppressed melanoma and colorectal cancer tumor growth (Fig. 7a–c, Supplementary Fig. S7c–e). As for combination therapy, immune checkpoint therapies have emerged in the effective treatment of melanoma, and it is reported that immune checkpoint inhibitors and BRAF-targeted therapies have an effect on unresectable advanced melanoma.^33–35^ The result of the immunosuppressive model for B16F10 CDX showed that immunity synergistically promoted the antitumor efficacy of erianin with dramatic increases of CD8a^+^, CD4^+^ T cells, and NK cells (Fig. 7e, Supplementary Fig. S8e, k and l), which provides a proposal for combination therapy of erianin and checkpoint inhibitor. We also observed erianin increased PD-L1 mRNA and protein expression, and it might enhance the activity of PD-1/PD-L1 antibody therapies (Supplementary Fig. S8b, d and f). For further confirmation, we conducted combination therapy of erianin and PD-L1 antibody in B16F10 CDX. Even though erianin or PD-L1 single therapy inhibits tumor growth, we did not observe the significant synergistic effect. In a phase 1 trial, the combination of atezolizumab and cobimetinib showed efficacy in end-stage solid tumors, which was not affected by KRAS/BRAF mutation status. Nevertheless, in another two phase 2 and phase 3 trials, they did not improve objective response rate (ORR) or overall survival (OS) in triple-negative breast cancer (TNBC) and metastatic colorectal cancer, respectively.^36,37^

In conclusion, erianin is a novel dual inhibitor of CRAF and MEK1/2 kinases, and suppresses constitutive activation of MAPK signaling pathway. Based on this study, it has shown activities for various BRAF V600E or KRAS mutant cancers, including melanoma and colorectal cancer in vitro and in vivo. Importantly, we provide a promising leading compound, which needs further clinical development, for the clinical therapy of melanoma and colorectal cancer.

## Materials and methods

### Cells and reagents

HEK293T cell line, melanoma A375, SK-MEL-2, SK-MEL-28 cell lines and the CRC cell line HCT116 were obtained from American Type Culture Collection. Normal Human Epidermal Melanocytes (NHEM) was purchased from PromoCell. All cells were incubated according to the recommended protocol. Erianin (purity ≥98%) was provided by Sichuan Weikeqi Biological Technology, Co., Ltd. (Chengdu, China). Vemurafenib (purity ≥99.80%), Dabrafenib (purity ≥99.97%), Encorafenib (purity ≥99.63%), Cobimetinib (purity ≥99.71%), Trametinib (purity ≥99.59%), Binimetinib (purity ≥99.55%), and LY3009120 (purity ≥99.01%) were purchased from MCE. C_18_H_18_O_5_ (purity ≥98%) was purchased from Sigma. Active BRAF (#B08-11BG), active BRAF V600E (#B08-12G), active RAF1 (EE) (#R01-13G), corresponding RAF substrate inactive MEK1 (#M02-14BG), as well as active MEK1 (#M02-10G), active MEK2 (#M03-10G), inactive ERK1 protein (#M29-14G), and inactive ERK2 protein (#M28-14U) were obtained from Signal Chem. Human RAF-1 protein (His & GST Tag) (#10657-H20B) was purchased from Sino Biological.

### Antibody list

Antibody of EGF Receptor (#2232), Phospho-BRAF (Ser445, #2696), CRAF (#53745), Phospho-c-Raf (Ser338, #9427), MEK1/2 (#4694), MEK1 (#12671), MEK1 (#2352), MEK2 (#9147), p-MEK1/2 (Ser217/221, #9154), ERK1/2 (#4695), p-ERK1/2 (Thr202/Tyr204, #4370), GST-Tag (#2624), PD-L1 (#13684) were obtained from Cell Signaling Technology (Danvers, MA), BRAF V600E (#A1137-25) from Biovision (San Francisco, USA), and BRAF (#ab33899), Ki67 (#ab16667) obtained from Abcam (Cambridge, USA). β-Actin (#sc47778) provided by Santa Cruz Biotechnology (Santa Cruz, USA), and anti-Flag antibody (#F1804) was obtained from Sigma–Aldrich (St. Louis, USA). FITC Rat Anti-Mouse CD45 (#553079), PerCP-Cy™5.5-CD8a (#551162), APC-NK-1.1 (#550627), PE-CD4 (#557308) obtained from BD (New Jersey, USA), and in vivo anti-mouse PD-L1 (#BE0101) obtained from BioXcel, USA.

### KinaseProfiler™ enzyme profiling assay

Enzyme profiling services were provided by KinaseProfiler™ (Eurofin, Brussels, Belgium). The concentration of erianin was 100 nM, and total 70 kinases were tested in this in vitro screening and profiling services (2 repetitions). For more details, refer to the manufacturer’s instructions (www.eurofinsdiscoveryservices.com).

### Cellular thermal shift assay

293 T cells were transfected with plasmids of MAP2K1/MAP2K1 (K97A, M146A) pcDNA3.1-3×Flag-C, MAP2K2/MAP2K1 (K101A, M150A) pcDNA3.1-3×Flag, RAF1/RAF1 (K375A) pcDNA3.1-3×Flag-C using lipo2000 for 48 h. After incubating with erianin for 4 h, the cells were collected into PCR tubes (100 μL) and incubated at a series of temperatures from 37 °C to 64 °C, with a gradient of 3 °C for 3 min. After being frozen by using liquid nitrogen and thawed on ice for twice, the supernatant was collected for the subsequent western blotting.

### ITC assay

The ITC experiment was performed on a MicroCal PEAQ-ITC. After cleaning the sample cell and needle, the MEK1 (38 μM), MEK2 (5 μM), or CRAF (10 μM) protein were carefully injected into the sample cell using a micro-syringe without any bubbles, and erianin (400 μM for MEK1 and MEK2, 150 μM for CRAF) was filled into a 40 μL titration syringe. All proteins and erianin were diluted with PBS (0.1% DMSO, pH = 7.4), and deionized water was injected into the reference cell as a heat balance control. After 19 times titrations of erianin into the sample cell at a constant rate of 150 s, the titration curve was fitted with nonlinear least squares estimation model using Microcal ORIGIN V7.0.

### SPR

Recombinant CRAF, MEK1 and MEK2 were coupled onto a CM5 chip (#BR-1005-30) via carboxyl groups on the dextran. After incubation, a series concentration of erianin (RAF inhibitor LY3009120 and MEK inhibitor cobimetinib were simultaneously added as positive control) flowed through the protein-CM5 system. The binding was tested and analyzed using Biacore T200 instrument.

### In vitro pull-down assay and ex vivo pull-down assay

Cell lysate (500 μg) or recombinant proteins (200 ng) were incubated with erianin-CNBr activated sepharose 4B overnight at 4 °C, and the protein-beads complex was washed four times by the use of rotational incubator to remove non-specific binding. Western blotting was developed to check the pull-down bands.

### Computational docking modeling

The computational docking model was constructed by the use of the Schrödinger Suite 2015 software programs (Schrödinger, 2015). The protein structures of MEK1 (PDB: 5eym),^38^ MEK2 (PDB:1s9i),^39^ and CRAF (PDB:3omv)^40^ were obtained from the protein data bank (PDB). MEK1, MEK2, and CRAF crystal structures were prepared according to the protein preparation wizard in Schrödinger Suite 2015.

### Molecular dynamics simulation

MEK1(PDB ID: 7m0z),^41^ MEK2 (PDB ID: 1s9i), and CRAF (PDB ID: 3omv) were selected for molecular dynamics (MD). Initially, the structures of erianin, atp and anp were quantum mechanically optimized using HF/6-31 G (d, p) method. After distribution of the electrostatic potential and determination of the atomic charges, erianin was docked into the potential binding pocket of the enzymes by the use of the AutoDock 4.2 program. The missing segments (residues 275–306 in 7m0z, residues 224–227 and 286–312 in 1s9i, and residues 493–504 in 3omv) were modeled using Alpha-Fold. After the ionizable residues were protonated, the complex structures were solvated in a rectangular box of TIP3P water molecules with a distance of 15 Å. Following addition of Na^+^ or Cl^−^, assignment of force field to the protein and a series of energy minimizations, run the simulation.

### Combination index assay

Combination index (CI) assay was used to measure drug-drug synergy based on median effect principle.^42^ Monotherapy drug (erianin or vemurafenib) was designed to different concentrations according to IC50 value, which was obtained from the cell proliferation assay. The synergism and antagonism (CI value) were determined and analyzed using CompuSyn 1.0.

### In vitro kinase assays and ADP-glo kinase assay

Inactive GST-MEK1 proteins were used as substrates with active BRAF (381-end) (BRAF 20 ng, MEK1 200 ng), BRAF V600E (416-end) (BRAF V600E 20 ng, MEK1 200 ng), or CRAF (306-end) kinase (CRAF 50 ng, MEK1 600 ng). Inactive GST-ERK1 or tag free ERK2 (400 ng) proteins were used as substrates with active GST-MEK1 full length or GST-MEK2 full length kinase (60 ng). After incubated with 100–400 μM ATP in kinase assay buffer for 30 min, the phosphorylation of the substrate/total substrate protein was detected by western blotting. For ADP-glo kinase assay, unconsumed ATP was depleted with the addition of ADP-Glo™. Followed by kinase detection reagent to convert ADP to ATP, consumption of ATP was detected to evaluate the kinase activity. For more details, refer to the manufacturer’s instructions of ADP-Glo™ Kinase Assay.

### Anchorage-independent cell growth assay

Three milliliters bottom agar (RPMI1640 or DMEM medium, 0.5% agar) was planted onto six-well plates, followed by cells (8 × 10^3^ cells/well) suspended in 1 mL top agar at 37 °C for 2–3 weeks. Colonies were captured (4 visual fields selected randomly for each well) by using the microscope, and then colony numbers or positive colony area was analyzed by Image-Pro Plus.

### Cytotoxicity and cell proliferation assay

For cytotoxicity test, normal NHDF (5000 cells/well) and NHEM cell line (3000 cells/well) were planted into 96-well plates. For cell proliferation assay, A375 (1500 cells/well), SK-MEL-28 (3000 cells/well), SK-MEL-2 (4000 cells/well), and HCT116 (1500 cells/well) were planted into 96-well plates, determined concentration of drug was added after culture for 24 h. The viability of the cells was detected within 72 h using MTT solution (5 mg/mL). The absorbance at 570/490 nm was quantified by the use of the microplate reader.

### Real-time PCR

Total RNA was obtained from cancer cells using TRIzol in the RNase-free context. After quantification with NanoDrop, cDNA was synthesized by using Fast Quant RT Kit. qPCR reaction system was prepared according to the SYBR Premix Ex Taq™ Kit (TAKARA, China). The expression of target gene in different cells was normalized to GAPDH. The primers for the PCR reaction are as follows: human PD-L1: 5′ -GGTGCCGACTACAAGCGAAT-3′ and 5′ -AGCCCTCAGCCTGACATGTC-3′; human GAPDH: 5′ -GACAAGCTTCCCGTTCTCAG-3′ and 5′ -GAGTCAACGGATTTGGTCGT-3′.

### Western blotting analysis

Protein was quantified using a BCA assay kit and then western blotting was conducted. For more details, refer to the previous paper.^43^

### Protein point mutations and purification

To identify the binding sites, protein point mutations were constructed using the Fast Mutagenesis System (Cat#FM111, Trans, China). MAP2K1 pET-28a (+), MAP2K2 pET-28a (+), and CRAF pCDNA3.1 3×flag plasmids were obtained from Youbao Biotechnology Company. After sequence identification and IPTG (1 mM) induction, the wild-type and mutant MEK1 and MEK2 proteins were purified with 6×His beads, and the wild-type and mutant CRAF were transfected into 293 T using lipo2000 reagent, followed by pull-down assay performed on these proteins.

### Lentiviral infection and overexpression

To establish stable protein knockdown cell lines, shRNA of CRAF, MEK1, and MEK2 oligonucleotide sequences were connected to enzyme digested pLKO.1 vector. To establish stable protein overexpression for phenotype, MAP2K1 pcDNA3.1-3×Flag-C, MAP2K2 pcDNA3.1-3×Flag, RAF1 pcDNA3.1-3×Flag-C were transfected into 293 T using lipo2000. To obtain stable protein overexpression for in vivo study, plasmids of MAP2K1 WT/MAP2K1 (K97A, M146A) pCDH-PURO-3×Flag, MAP2K2 WT/MAP2K2 (K101A, M150A) pCDH-PURO-3×Flag and RAF1 WT/RAF1 (K375A) pCDH-PURO-3×Flag were transfected into 293 T using jetPRIME. After lentivirus was collected, 8 μg/mL polybrene was chosen to infect A375 cell line, followed by the selection process using 2 μg/mL puromycin for more than 72 h. For detailed protocols, refer to the prior study.^44^

### CRISPR/Cas9 knockout cell lines

Deplete MEK1/2 or CRAF using the CRISPR/Cas9 system. sgRNA of CRAF, MEK1, and MEK2 oligonucleotide sequences were connected to enzyme digested pLKO.1 vector. After vectors package in 293 T and virus harvest, 8 μg/mL polybrene was chosen to infect A375 and SK-MEL-2 cells, followed by the selection process using 2 μg/mL puromycin for more than 72 h. The knockout efficacy was determined using Western blot.

### Cell-derived xenograft (CDX) and patient-derived xenograft (PDX) mouse model

The animal experiments were performed according to the guidelines approved by the Zhengzhou University Institutional Animal Care and Use Committee. A375 cell line (1 × 10^7^ cell/mouse), SK-MEL-28 cell line (5 × 10^6^ cell/mouse), SK-MEL-2 cell (5 × 10^6^ cell/mouse), and HCT116 cell line (1 × 10^7^ cell/mouse) were injected subcutaneously into the NOD-SCID mice (Vital River Labs, Beijing, China). For the melanoma PDX mice model, the tumor was taken after surgical operation and implanted into the NPG (Vital River Labs, Beijing, China) mice under a sterile environment. For the colorectal cancer PDX mice model, NOD-SCID mice were selected as carriers for tumor growth. The measurement of tumor size was performed twice a week using vernier caliper and the calculation of tumor volume was done according to tumor volume (mm^3^) = length × width × height × 0.52.

### Flow cytometry

Peripheral blood or spleen lymphocytes (CD8a^+^, CD4^+^, and NK cell) in B16F10 CDX model after treated with erianin were quantified using BD FACSAria^TM^ III cell sorter. Peripheral blood (100 μL) with heparin was lysed with erythrocyte lysate (#555899, BD) in the dark for 15 min. After 30 min of incubation with antibodies at 4 °C and washing with staining buffer, the cell was resuspended using PBS for analysis.

### Pharmacokinetic assay

For pharmacokinetic study of erianin, LC-MS/MS was implemented to monitor the plasma concentration over times and associated parameters. Chromatographic separation was obtained on an ZORBAX SB-C18 column (3.5 μm, 3.0 × 100 mm). 85% acetonitrile was selected as the mobile phase. The conditions of ESI source were optimized as follows: Spray voltage 4000 V; nitrogen for dying with a flow rate of 11 L/min. Detect the selected reaction monitoring mode for quantification, *m/z* 315.0 > 180.8 for IS and *m/z* 319.1 > 150.9 for erianin, respectively. 2′-Hydroxy-4,4′,6′-trimethoxychalcone (500 ng/mL) was selected as internal standard. After the validation of standard curves (0.1, 1, 5, 10, 50, 100, 500, 800, and 1200 ng/mL for oral; 10, 50, 100, 500, 800, 1200, 2400, 4800, 9600 ng/mL for intravenous) and methods including selectivity, linearity, sensitivity, precision and stability, erianin was given to Kunming mice through oral gavage (50 mg/kg, *n* = 6) or tail vein injection (25 mg/kg, *n* = 4). A series of time points including 0, 0.083, 0.25, 0.5, 0.75, 1, 2, 3, 4, and 8 h were set for blood extraction. The pharmacokinetic parameters were analyzed using DAS 2.0. Absolute bioavailability (F%) = (AUC_oral_ × D_iv_)/(AUC_iv_ × D_oral_) × 100%.

### Statistical analysis

Data were presented in the form of mean ± standard deviation (SD). Student’s *t*-test (within two groups), one-way ANOVA (more than two groups) and Kaplan–Meier analysis (survival rate of tumor-bearing mice) were performed by the use of GraphPad Prism 7.0. *p* ≤ 0.05 was considered statistically significant through the whole study.

## Supplementary information

Supplementary Materials for Erianin suppresses constitutive activation of MAPK signaling pathway by inhibition of CRAF and MEK1/2

Supplementary Table S1

Supplementary Table S2

Supplementary raw data

## Data Availability

The authors declare that the data supporting the findings of this study are available within the paper and its Supplementary materials.
