# Supplementary material for: Erianin suppresses constitutive activation of MAPK signaling pathway by inhibition of CRAF and MEK1/2
Source: Signal Transduct Target Ther. 2023 Mar 6;8:96. doi: 10.1038/s41392-023-01329-3 (PMC9986241; doi:10.1038/s41392-023-01329-3)
Supplement: Supplementary file 1 — Supplementary Materials for Erianin suppresses constitutive activation of MAPK signaling pathway by inhibition of CRAF and MEK1/2 [file 41392_2023_1329_MOESM1_ESM.docx]

Supplementary Materials for

**Erianin suppresses constitutive activation of MAPK signaling pathway by inhibition of CRAF and MEK1/2**

Penglei Wang, Xuechao Jia, Bingbing Lu, Han Huang, Jialin Liu, Xuejiao Liu, Qiong Wu, Yamei Hu, Pan Li, Huifang Wei, Tingting Liu, Dengyun Zhao, Lingwei Zhang, Xueli Tian, Yanan Jiang, Yan Qiao, Wenna Nie, Xinli Ma, Ruihua Bai, Cong Peng, Zigang Dong^✉^, and Kangdong Liu^✉^

Penglei Wang, Xuechao Jia and Bingbing Lu contributed equally to this work.

Correspondence: Kangdong Liu ([kdliu@zzu.edu.cn](mailto:kdliu@zzu.edu.cn)) or Zigang Dong ([dongzg@zzu.edu.cn](mailto:dongzg@zzu.edu.cn))

**This PDF file includes:**

Figures S1 to S9

Supplementary Table S1 to S2 (attached as single excel files)


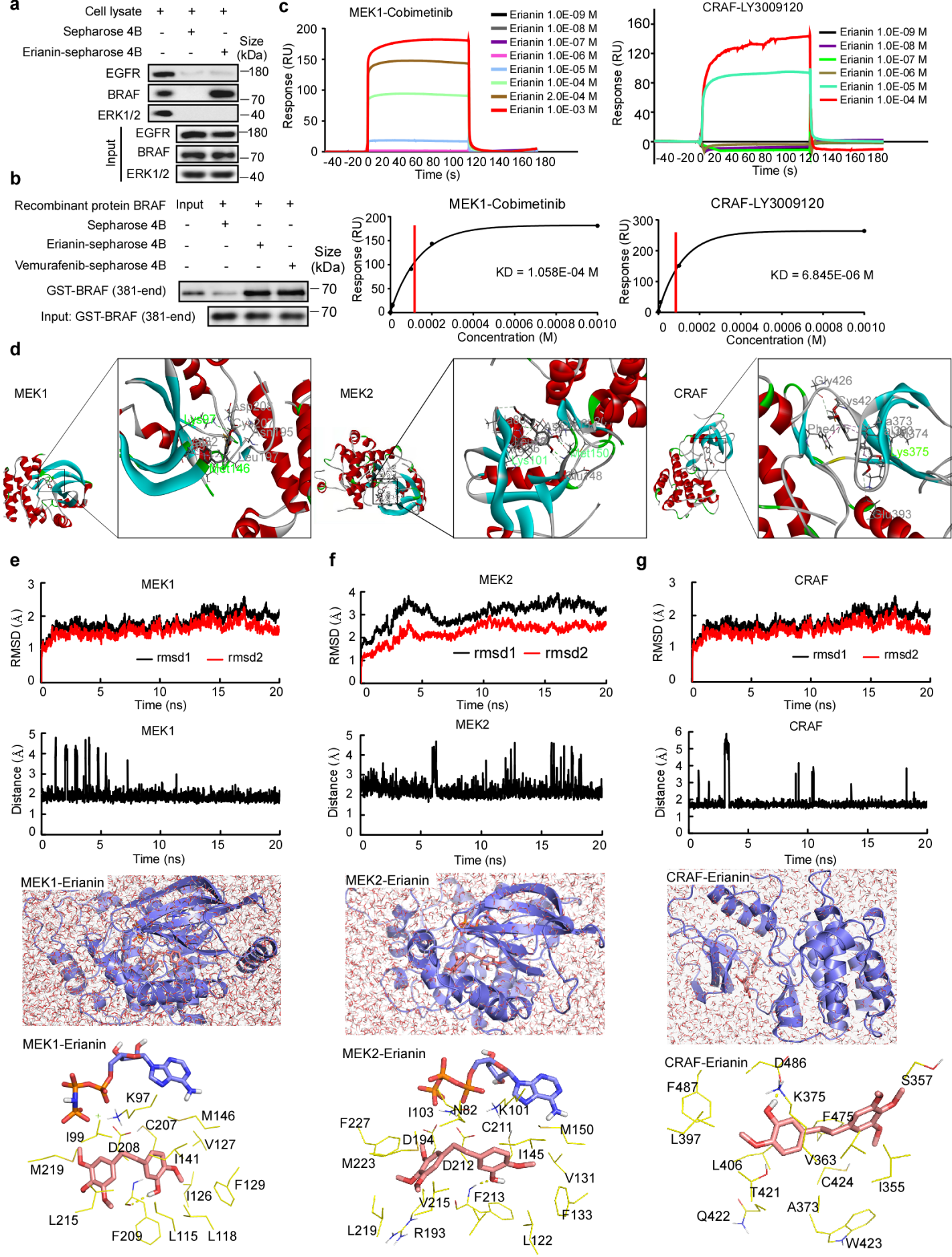


**Figure S1. Discovery of a direct CRAF and MEK1/2 inhibitor erianin. a** Erianin binding with other MAPK members (EGFR, BRAF and ERK1/2) present in A375 cell lysates was determined using a erianin-conjugated Sepharose 4B bead pull-down assay. The band was visualized by Western blotting. **b** Erianin binding with human recombinant BRAF (381-end) protein was evaluated using a erianin-conjugated bead pull-down assay. The band was visualized by Western blotting. **c** SPR was performed using human recombinant CRAF (306-end) , MEK1 full length and corresponding positive controls (LY3009120 and cobimetinib). The equilibrium dissociation constant (KD) was evaluated according to the response-concentration curve. **d** Global computational docking between erianin and MEK1, MEK2 or CRAF. **e, f, g** Molecular dynamics simulations were performed to predict the binding modes of erianin with MEK1, MEK2, and CRAF. The figures show the root mean square deviation (RMSD) values during 20 ns MD simulations (top panel), key hydrogen bond distance (middle panel), representative binding complex structure and detailed binding mode between erianin and MEK1, MEK2, and CRAF, respectively (bottom panel).


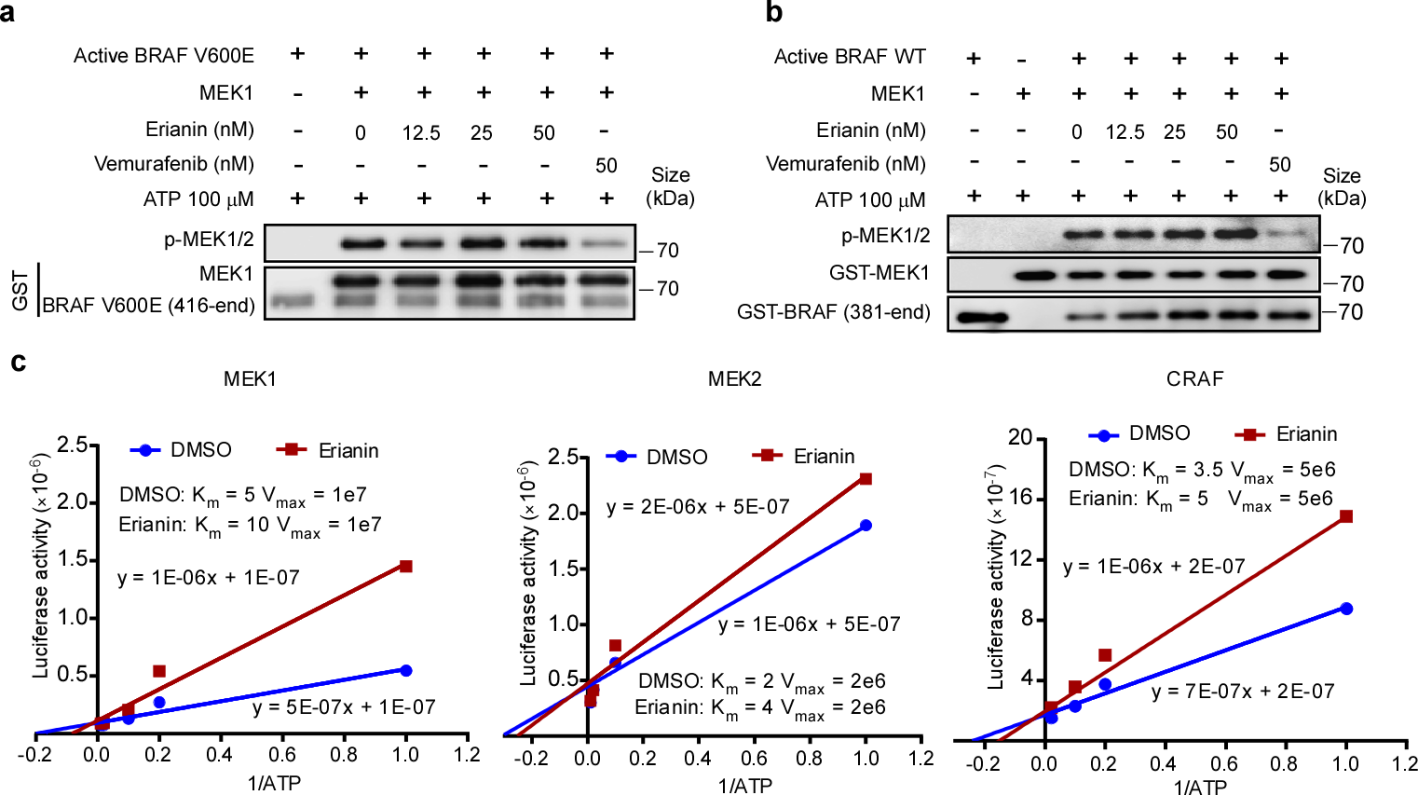


**Figure S2. Additional data to erianin inhibiting MAPK signaling pathway through suppressing CRAF and MEK1/2 but not BRAF kinase activity.** **a, b** Effect of erianin on BRAF V600E (416-end) and BRAF WT (381-end) kinase activity. The phosphorylation of MEK1/2 (Ser217/221) was detected by Western blotting. **c** Enzyme kinetics assay between erianin and MEK1 full length, MEK2 full length or CRAF (306-end). Enzyme kinetics parameters including V_max_ and K_m_ were obtained using the Michaelis double-reciprocal curve equation.


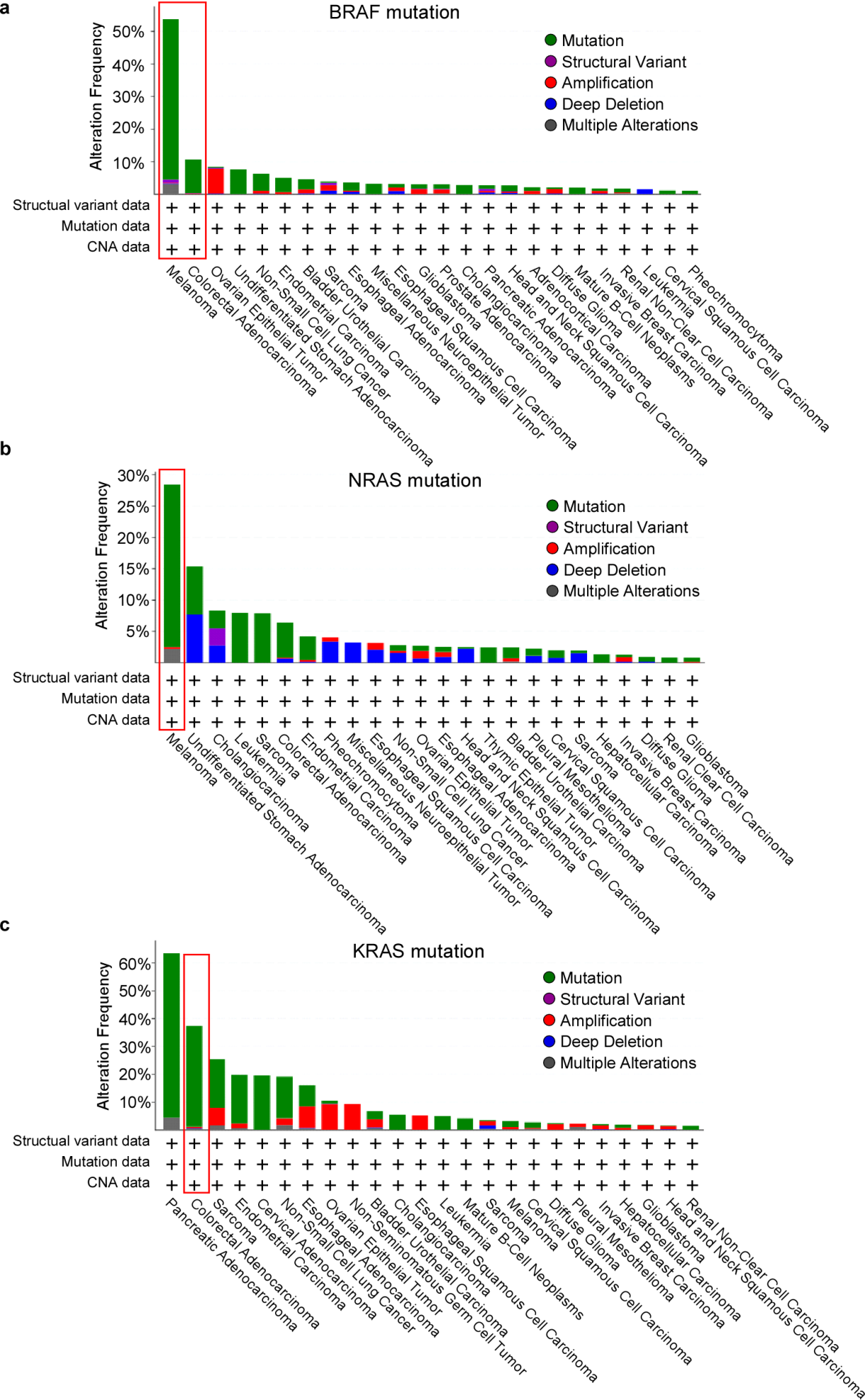


**Figure S3. a-c. Mutations of BRAF, NRAS and KRAS in multiple cancer types.** Genetic alterations of BRAF (**a**), NRAS (**b**) and KRAS (**c**) were obtained from TCGA database, and statistical analysis was performed on the cBioPortal platform.


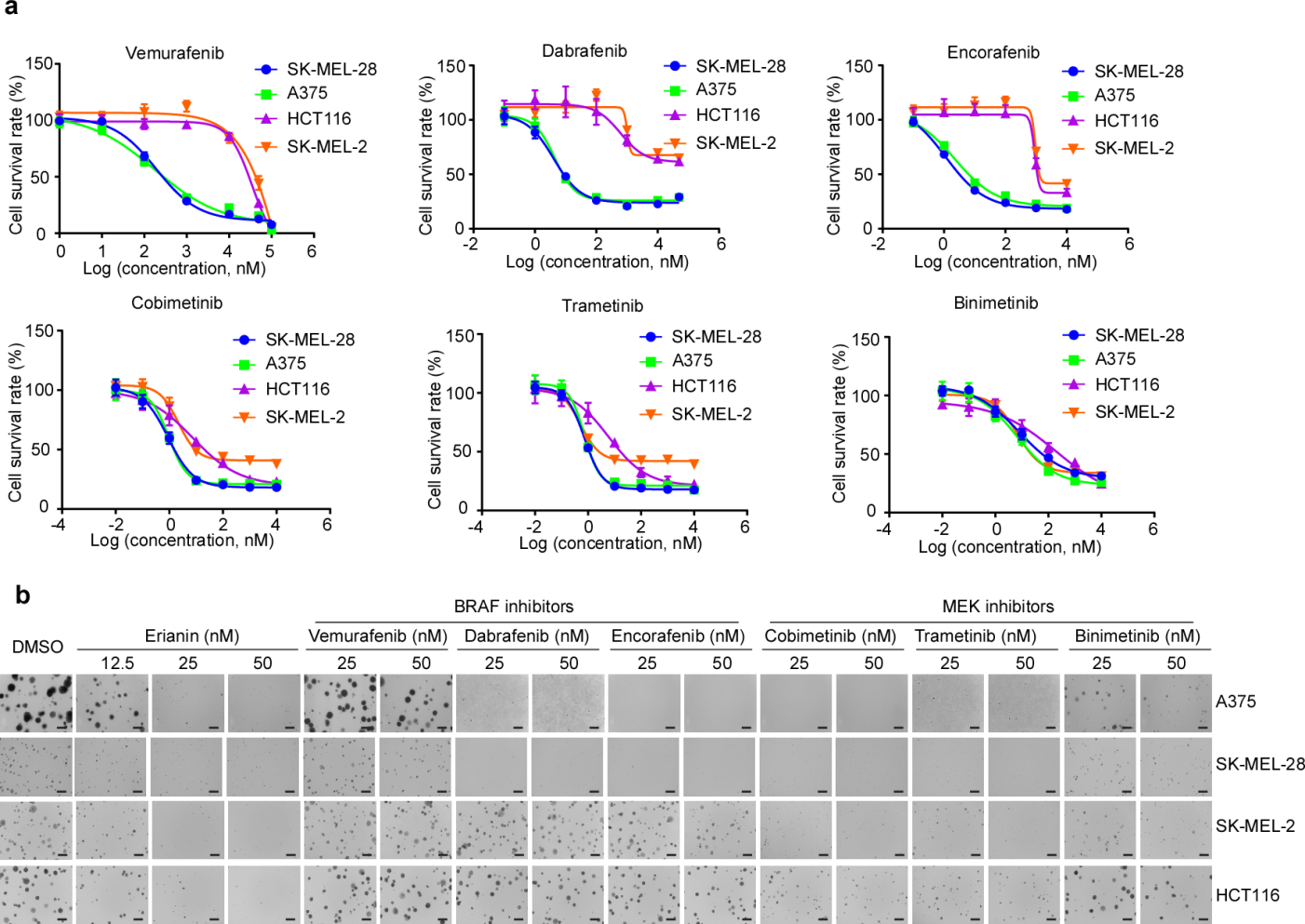


Figure S4. Additional data to erianin inhibiting proliferation in BRAF V600E or RAS mutant cell lines. a Representative dose–response curves of three BRAF inhibitors (vemurafenib, dabrafenib and encorafenib) and three MEK inhibitors (cobimetinib, trametinib and binimetinib) in SK-MEL-2 (NRAS mut), HCT116 (KRAS mut), A375 (BRAF V600E) and SK-MEL-28 (BRAF V600E) cell lines. The concentrations are transformed to Log10 values; the Y axis shows the corresponding relative cell viability. b The effect of erianin on anchorage-independent growth of the above cells was evaluated. Data were shown as mean ± S.D. Scale bars: 400 μm.


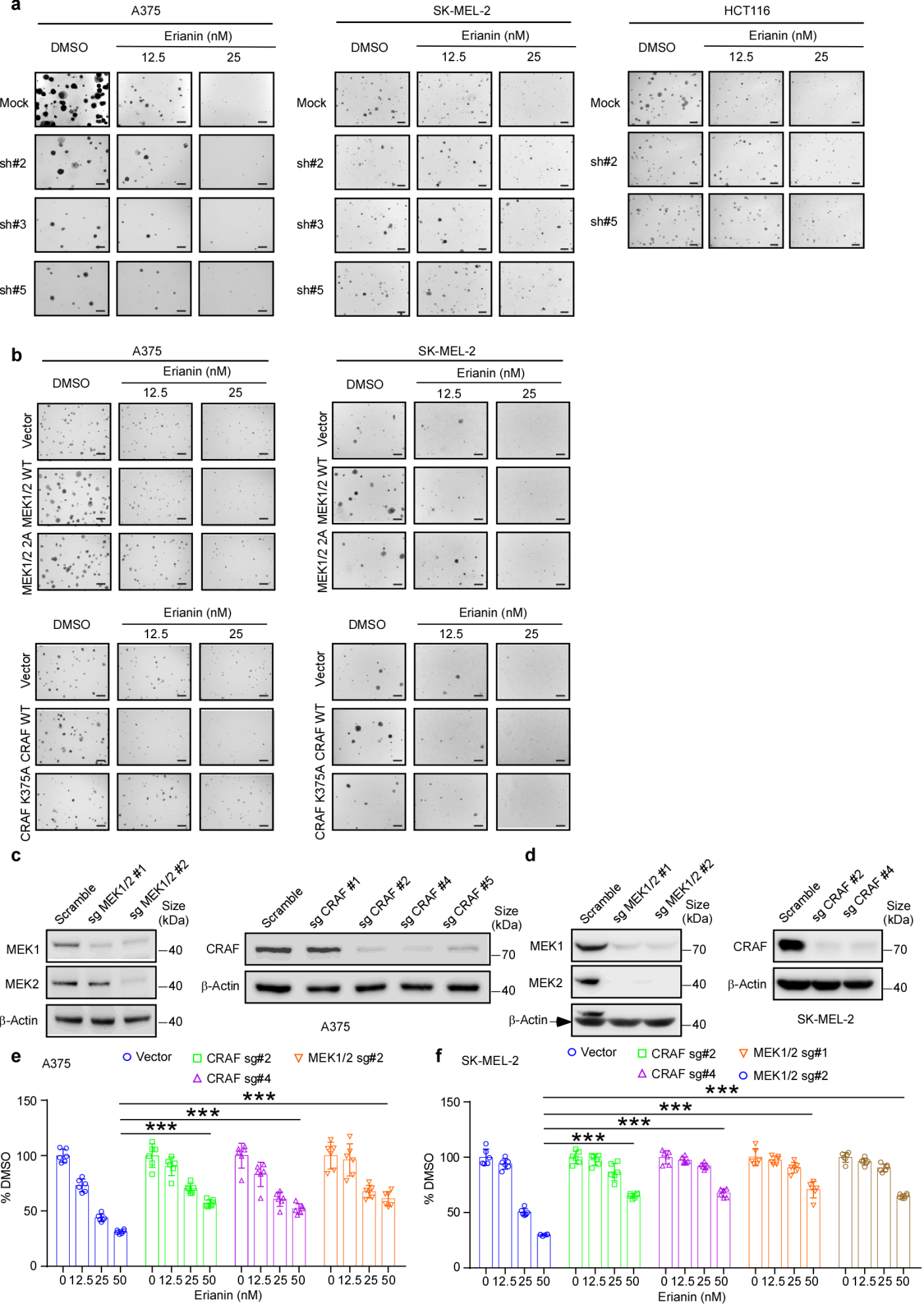


Figure S5. Depletion of CRAF and/or MEK1/2 can abolish the efficiency of erianin. a The effect of erianin on anchorage-independent growth was assessed in A375, SK-MEL-2 and HCT116 cells transfected with plasmid of shRNA-mock or shRNA-CRAF/MEK1/2. b Anchorage-independent growth of erianin in A375 and SK-MEL-2 cell lines (knock down endogenous MEK1/2 or CRAF, followed by transfecting plasmid of pcDNA3.1-3xflag-MEK1/2 WT/2A or CRAF WT/K375A). Scale bars: 400 μm. c, d Depletion of MEK1/2 or CRAF in A375 and SK-MEL-2 cell lines using the CRISPR/Cas9 system. e, f MTT assay of erianin in A375 and SK-MEL-2 after MEK1/2 or CRAF knock out.


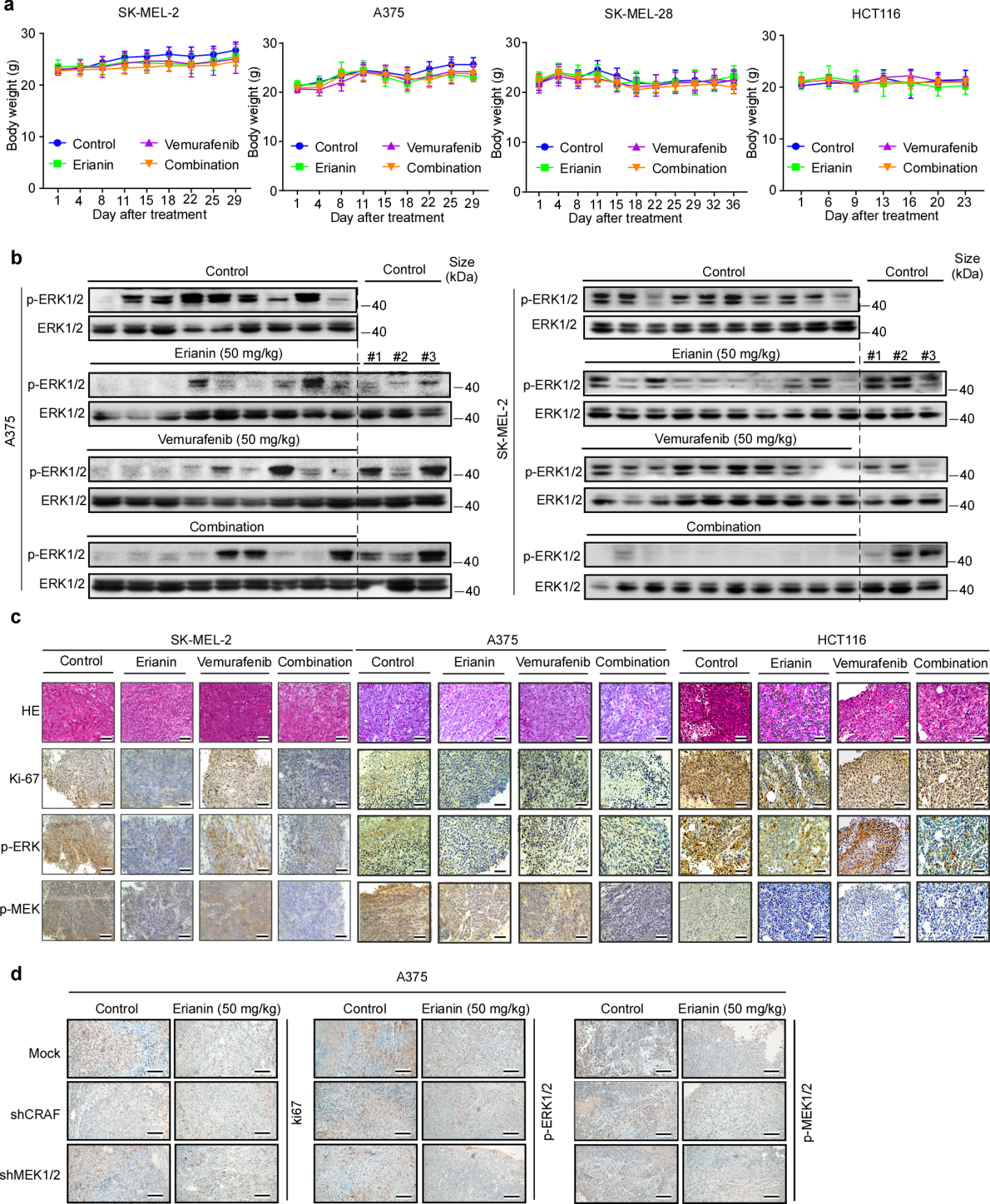


**Figure S6. Erianin suppresses MAPK signaling pathway in CDX model through inhibiting both CRAF and MEK1/2. a** Changes of body weight after drug administration in melanoma and CRC CDX mice model over time. **b** Western blotting shows the effect of erianin on phospho-ERK1/2 and T-ERK1/2 in A375 (BRAF V600E mutation) and SK-MEL-2 (NRAS mutation) CDX tumor tissues. These samples were prepared from all CDX tumor tissues from each mouse of each treatment group and each blot showed one sample. Three samples randomly selected from control group were set as negative control. **c** IHC staining (ki-67, p-ERK1/2 and p-MEK1/2) in A375, SK-MEL-2 and HCT116 CDX tumor tissues. Scale bars: 50 μm. **d** IHC staining (ki-67, p-ERK1/2 and p-MEK1/2) in A375 shCRAF or shMEK1/2 CDX tumor tissues.


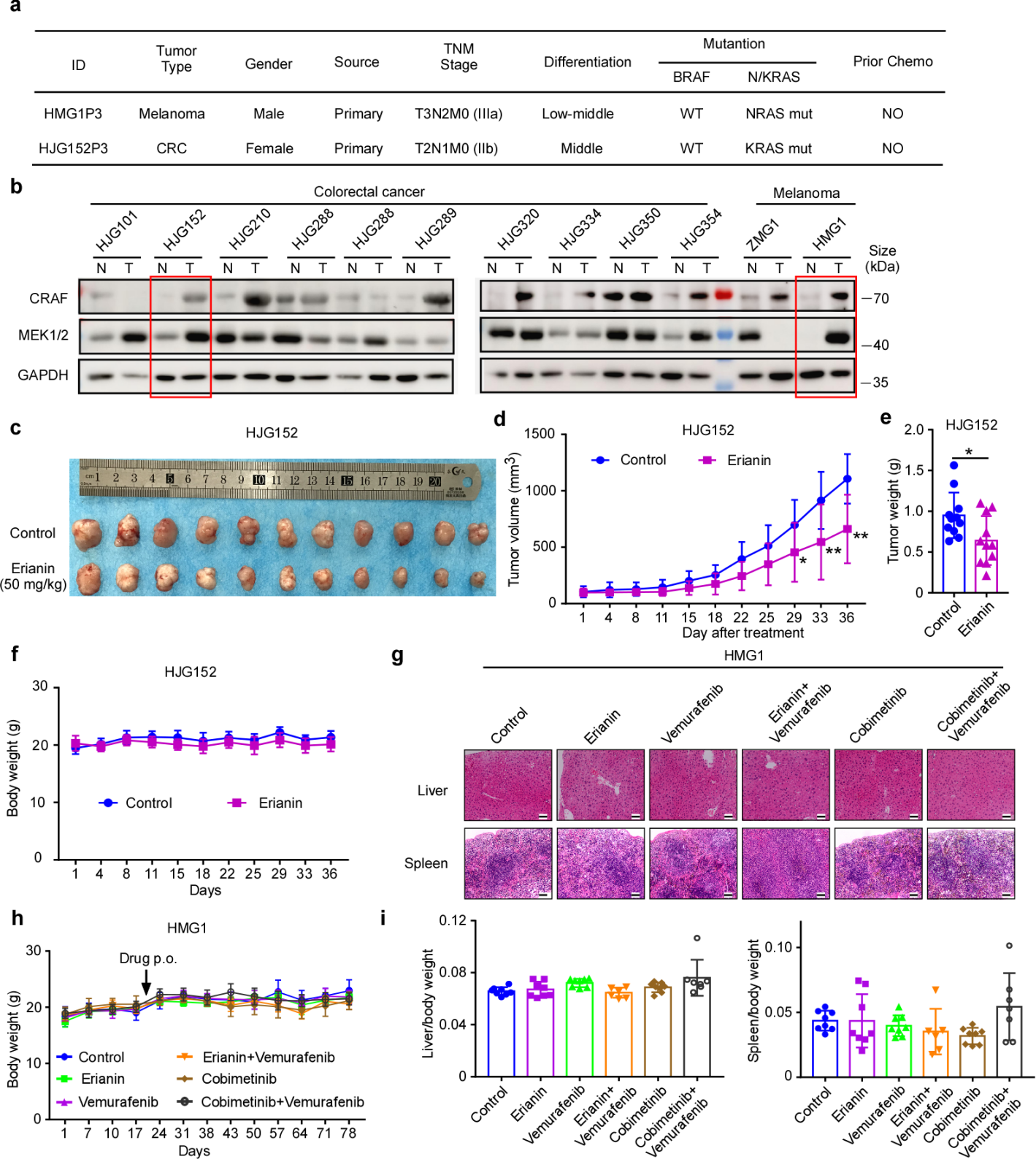


Figure S7. Additional data to erianin exerting anti-tumor efficacy in melanoma and colorectal cancer in vivo. a Patient information of melanoma and colorectal cancer (CRC) PDX model. b Expression of CRAF and MEK1/2 in adjacent tissues (nevus for melanoma) and tumor tissues of colorectal cancer and melanoma. c The photographs of tumors from CRC PDX model. d. Tumor volume of (c). e Tumor weight of (c). f, h Changes of body weight after drug administration in melanoma and CRC PDX model. g, i HE staining of liver and spleen in melanoma PDX model. Scale bars: 200 μm.


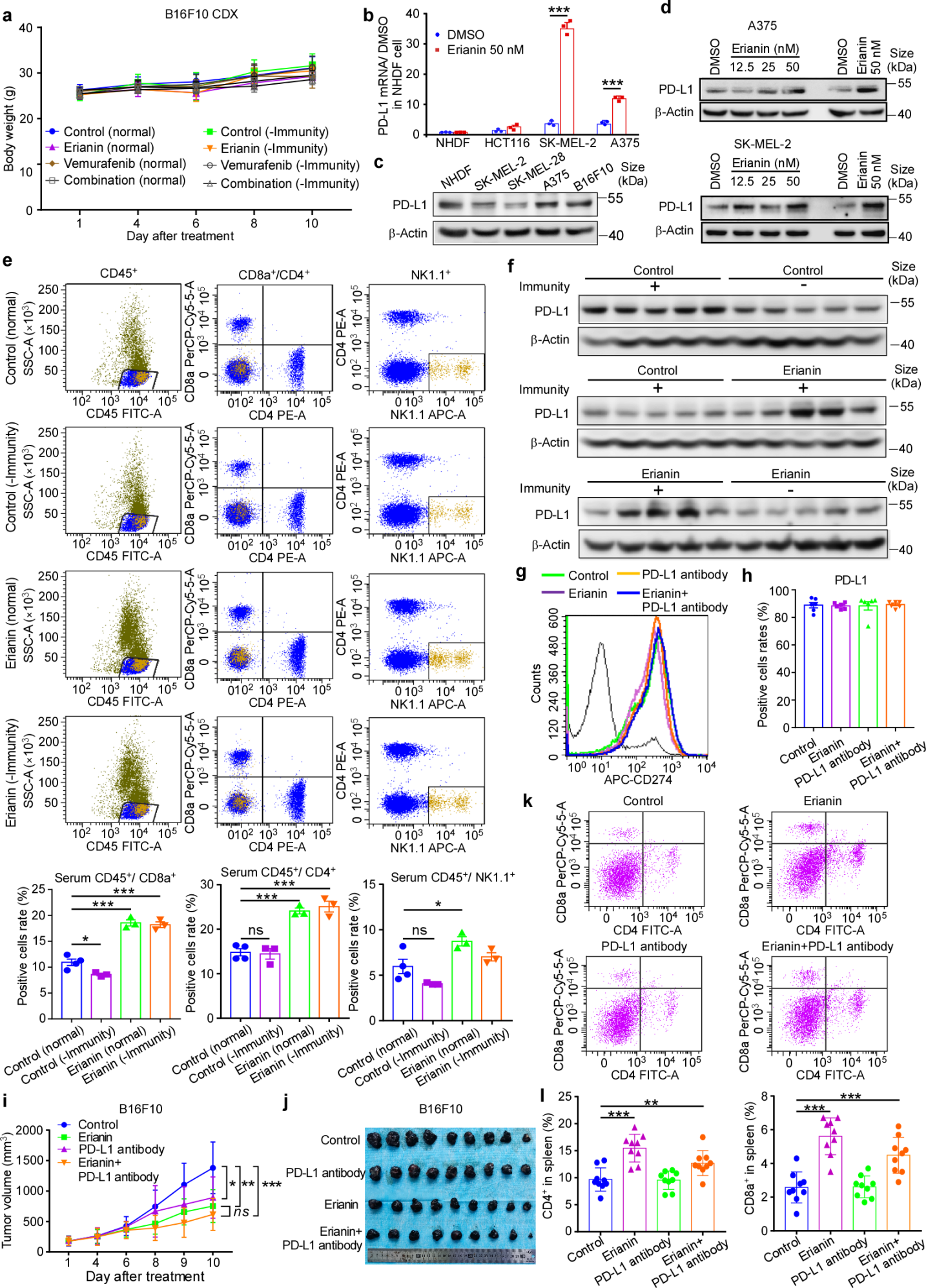


Figure S8.  Erianin promotes immune response without decreasing PD-L1 expression. a Changes of body weight after drug administration in B16F10 cell xenograft model. b PD-L1 mRNA expression after treatment with erianin in A375, SK-MEL-2 and HCT116 cell lines. Student’s *t*-test. *: *p* < 0.05; **: *p* < 0.01. c Expression of PD-L1 protein in different cell lines. d PD-L1 protein expression after treatment with erianin in A375, SK-MEL-2 cell lines. e Immune cell expression (CD8a^+^, CD4^+^ and NK cells) after erianin treatment. f PD-L1 protein expression after drug administration in B16F10 cell xenograft model. g, h Positive rate of PD-L1 in B16F10 CDX. i Tumor volumes curve over times in B16F10 CDX. j The photographs show tumors in B16F10 CDX mice. k Surface staining of CD4^+^ and CD8a^+^ in B16F10 CDX by Flow Cytometry. l Statistical results of (k). Data were presented as mean ± S.D. One-way ANOVA test. *: *p* < 0.05; **: *p* < 0.01; ***: *p* < 0.001.


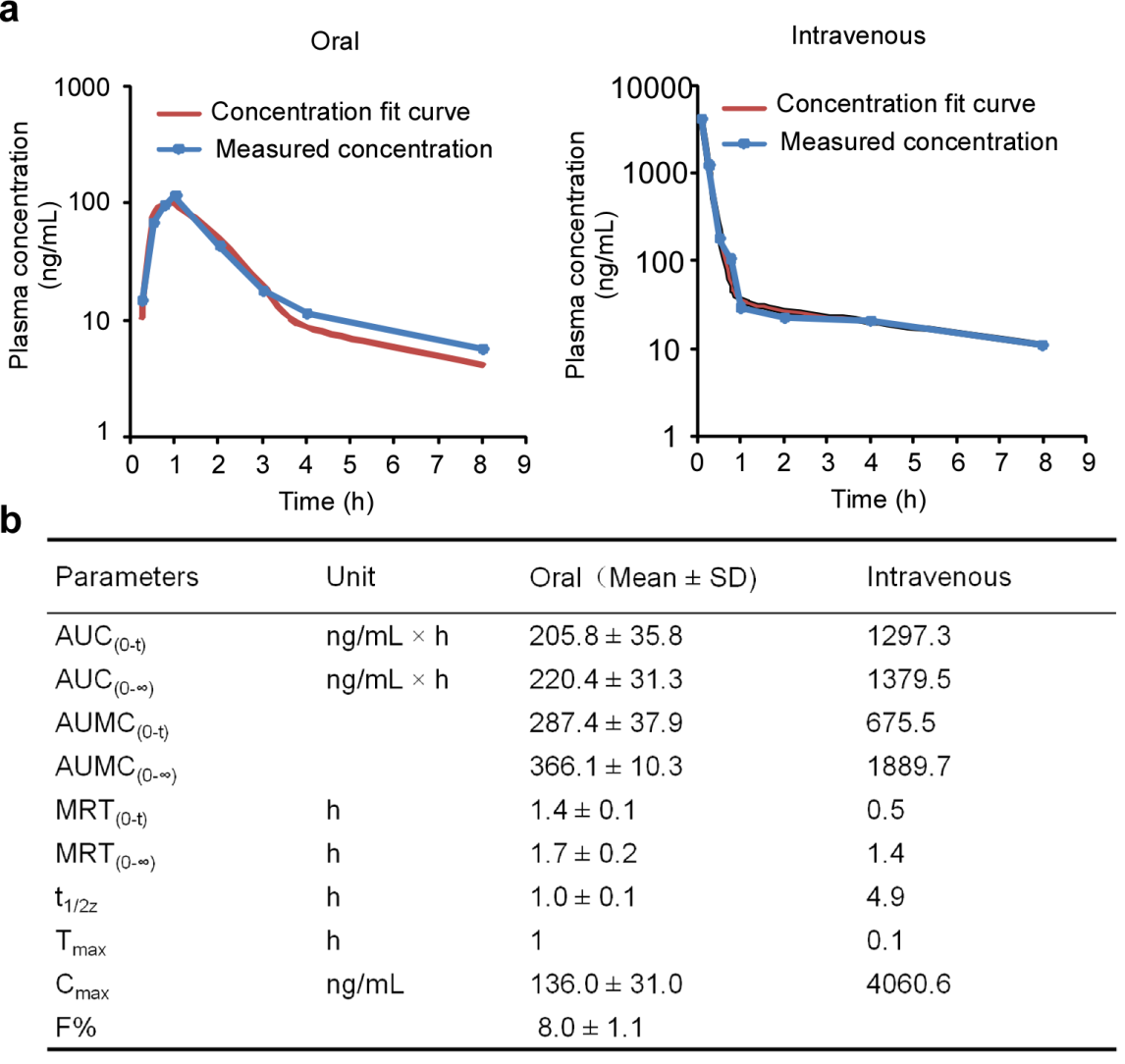


Figure S9 Pharmacokinetics assay of erianin. a Drug-time curve of erianin through oral and intravenous administration. b Pharmacokinetic parameters of erianin through oral and intravenous administration.
