# Supplementary material for: Erianin suppresses constitutive activation of MAPK signaling pathway by inhibition of CRAF and MEK1/2
Source: Signal Transduct Target Ther. 2023 Mar 6;8:96. doi: 10.1038/s41392-023-01329-3 (PMC9986241; doi:10.1038/s41392-023-01329-3)

## Fig. 1b

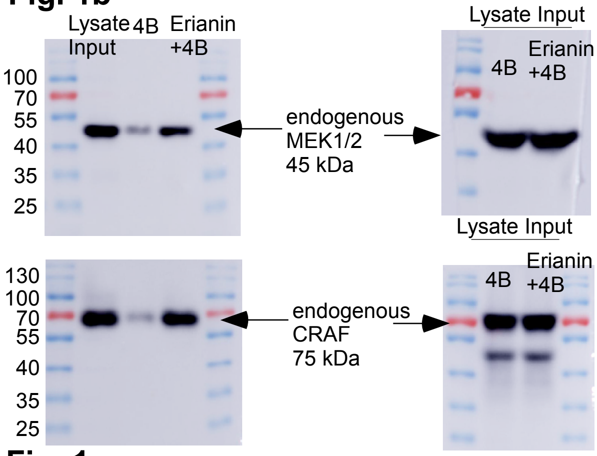


## Fig. 1c

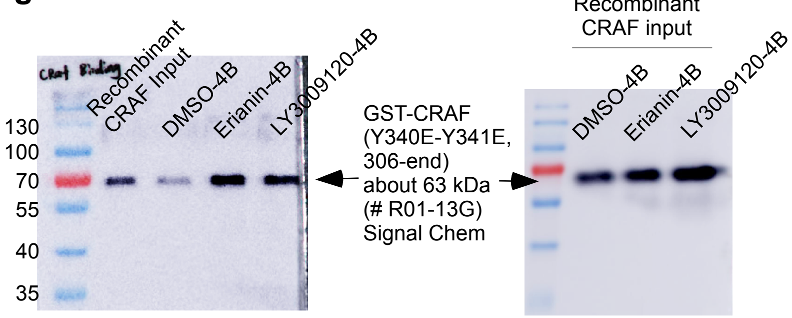


## Supplementary Fig. S1b

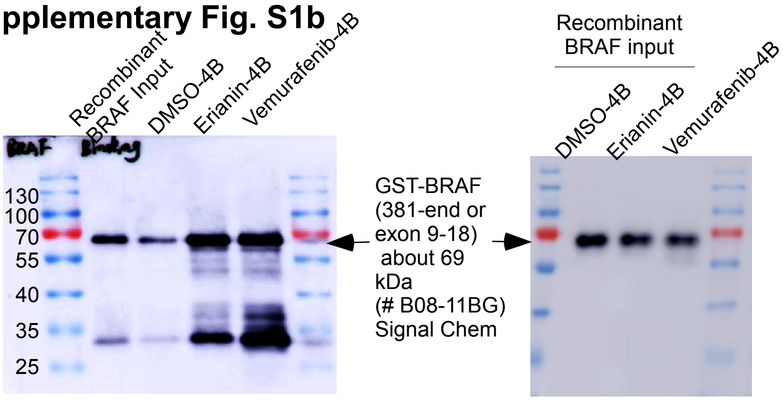


## Fig. 1c

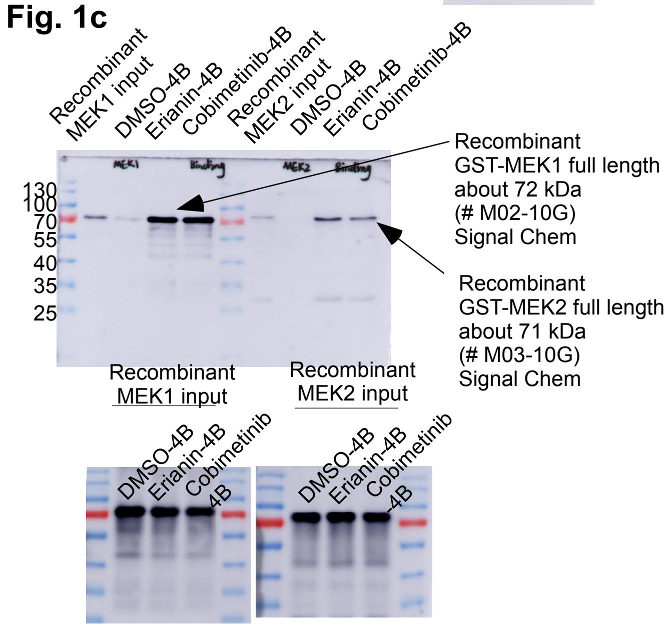


## Supplementary Fig. S1a

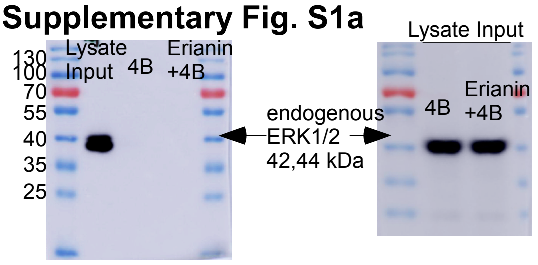


## Supplementary Fig. S1a

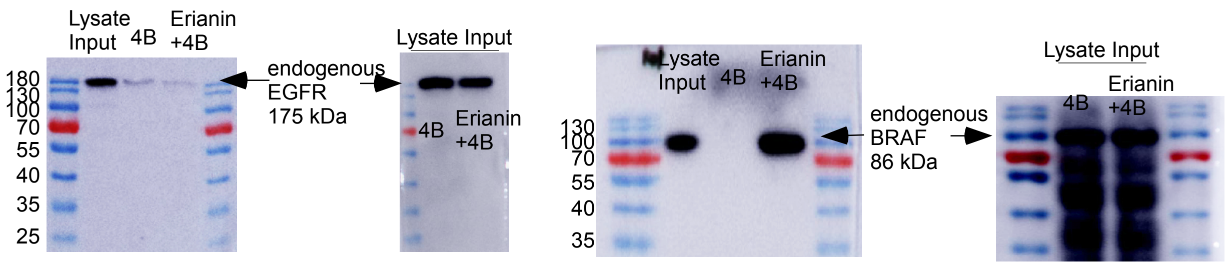


## Fig.1h

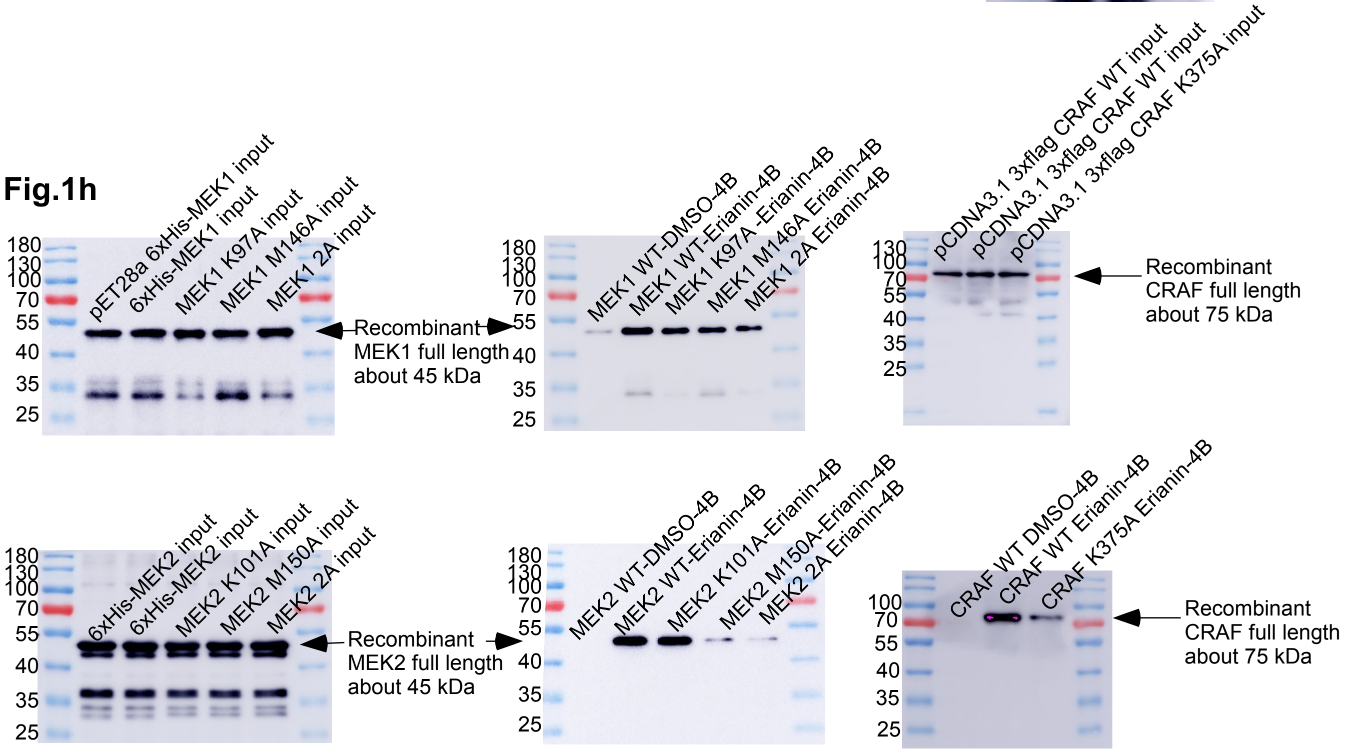

Figure 1g

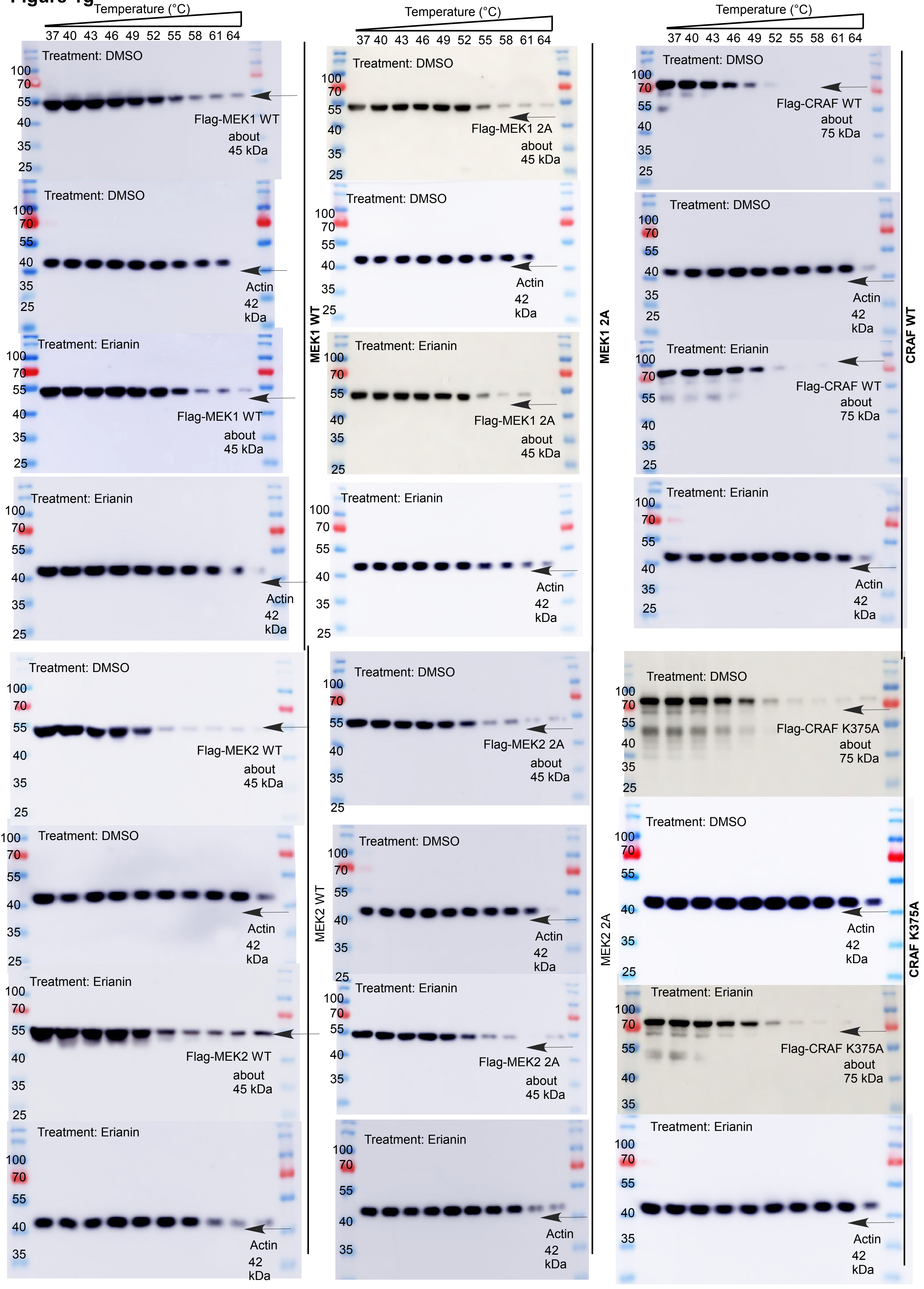

Temperature (°C)
37 40 43 46 49 52 55 58 61 64
Treatment: DMSO
Flag-MEK1 WT
about
45 kDa
Actin
42
kDa
Treatment: Erianin
MEK1 WT
Flag-MEK1 2A
MEK1 2A
Flag-CRAF WT
about
75 kDa
CRAF WT
Flag-MEK2 WT
MEK2 WT
Flag-MEK2 2A
MEK2 2A
Flag-CRAF K375A
CRAF K375A
100
70
55
40
35
25

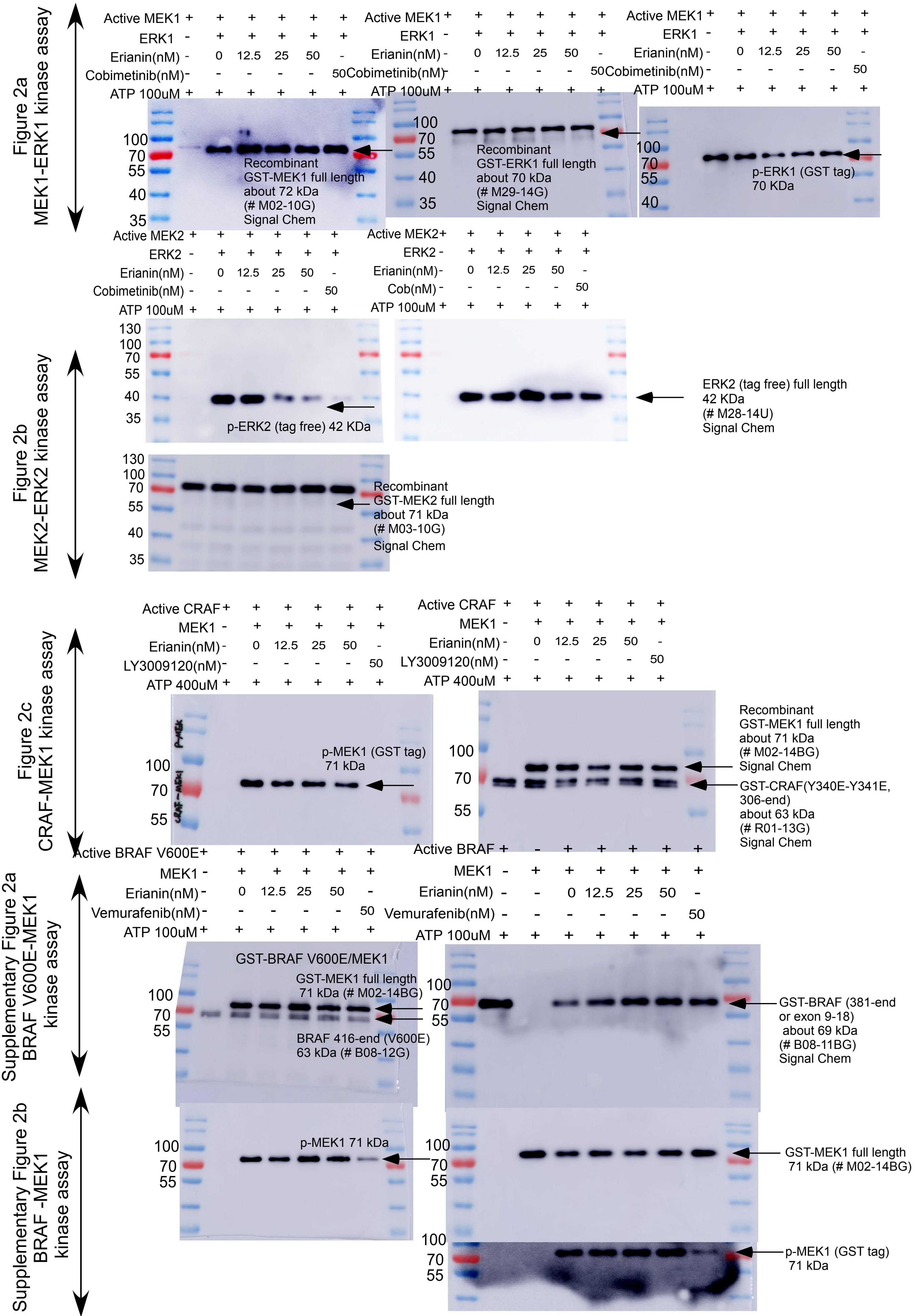
Figure 2a
MEK1-ERK1 kinase assay
Active MEK1 + + + + + +
ERK1 - + + + + +
Erianin(nM) - 0 12.5 25 50 -
Cobimetinib(nM) - - - - - 50
ATP 100uM + + + + + +
100
70
55
40
35
Recombinant
GST-MEK1 full length
about 72 kDa
(# M02-10G)
Signal Chem
Recombinant
GST-ERK1 full length
about 70 kDa
(# M29-14G)
Signal Chem
p-ERK1 (GST tag)
70 KDa
Figure 2b
MEK2-ERK2 kinase assay
Active MEK2 + + + + + +
ERK2 - + + + + +
Erianin(nM) - 0 12.5 25 50 -
Cobimetinib(nM) - - - - - 50
Cob(nM) - - - - - 50
ATP 100uM + + + + + +
130
p-ERK2 (tag free) 42 KDa
ERK2 (tag free) full length
42 KDa
(# M28-14U)
Signal Chem
Recombinant
GST-MEK2 full length
about 71 kDa
(# M03-10G)
Signal Chem
Figure 2c
CRAF-MEK1 kinase assay
Active CRAF + + + + + +
MEK1 - + + + + +
Erianin(nM) - 0 12.5 25 50 -
LY3009120(nM) - - - - - 50
ATP 400uM + + + + + +
p-MEK1 (GST tag)
71 kDa
Recombinant
GST-MEK1 full length
about 71 kDa
(# M02-14BG)
Signal Chem
GST-CRAF(Y340E-Y341E,
306-end)
about 63 kDa
(# R01-13G)
Signal Chem
Supplementary Figure 2a
BRAF V600E-MEK1
kinase assay
Active BRAF V600E + + + + + +
MEK1 - + + + + +
Erianin(nM) - 0 12.5 25 50 -
Vemurafenib(nM) - - - - - 50
ATP 100uM + + + + + +
GST-BRAF V600E/MEK1
GST-MEK1 full length
71 kDa (# M02-14BG)
BRAF 416-end (V600E)
63 kDa (# B08-12G)
Active BRAF + - + + + + +
MEK1 - + + + + + +
Erianin(nM) - - 0 12.5 25 50 -
Vemurafenib(nM) - - - - - - 50
ATP 100uM + + + + + + +
GST-BRAF (381-end
or exon 9-18)
about 69 kDa
(# B08-11BG)
Signal Chem
Supplementary Figure 2b
BRAF -MEK1
kinase assay
p-MEK1 71 kDa
GST-MEK1 full length
71 kDa (# M02-14BG)
p-MEK1 (GST tag)
71 kDa

## Figure 2h

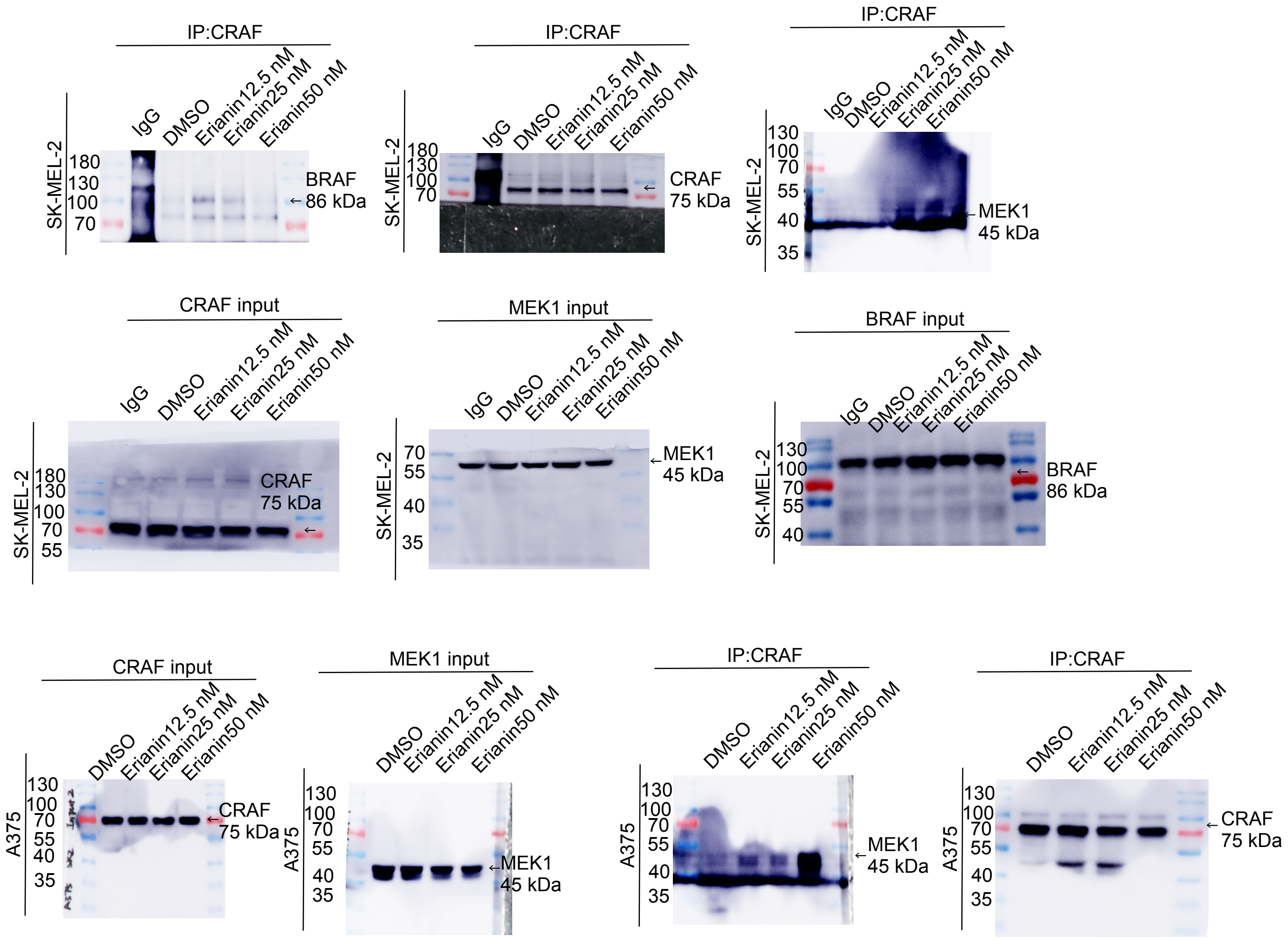


## Figure 2i

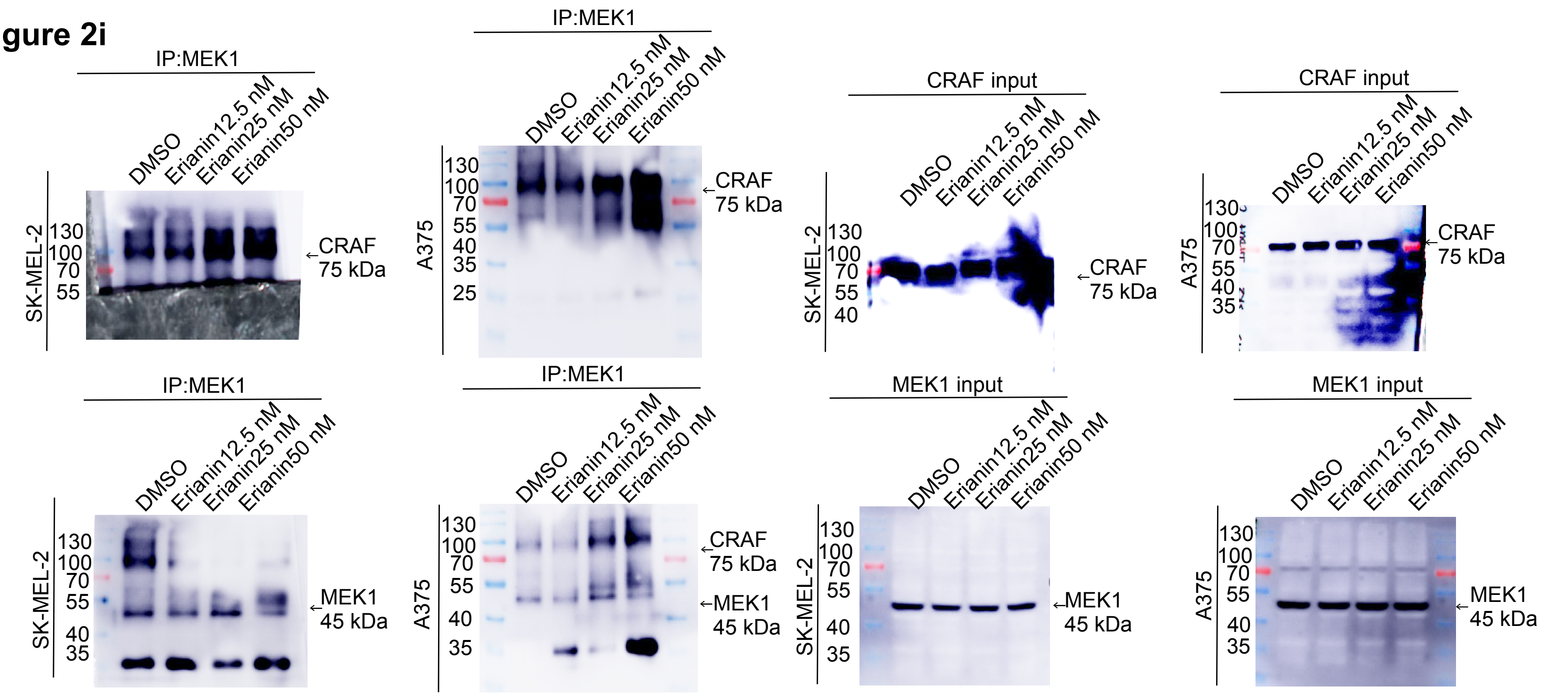

# Figure 2j

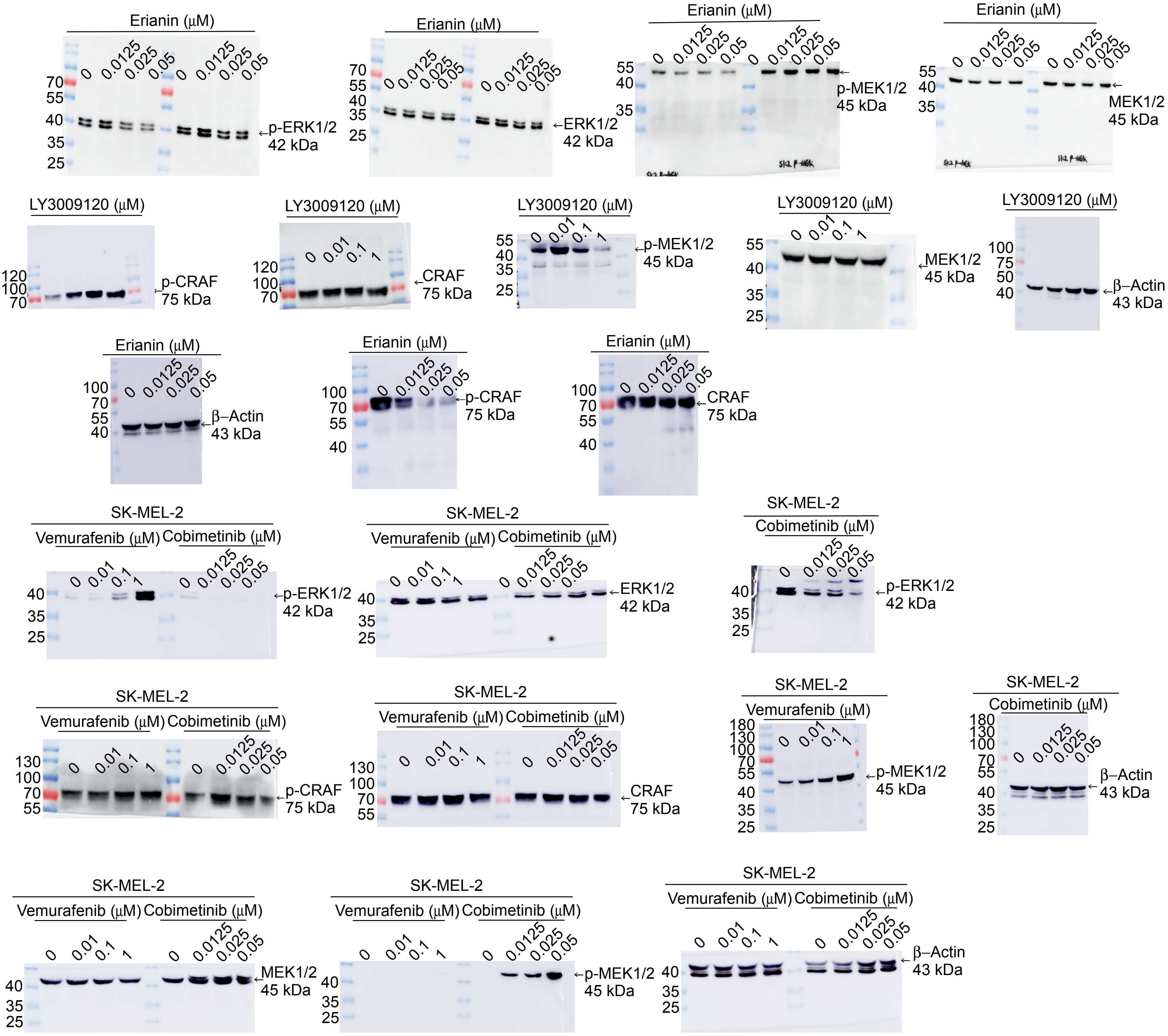

Erianin (μM)
0 0.0125 0.025 0.05 0 0.0125 0.025 0.05
70 55 40 35 25
p-ERK1/2 42 kDa
ERK1/2 42 kDa
p-MEK1/2 45 kDa
MEK1/2 45 kDa
LY3009120 (μM)
0 0.01 0.1 1
120 100 70
p-CRAF 75 kDa
CRAF 75 kDa
β–Actin 43 kDa
SK-MEL-2
Vemurafenib (μM)
Cobimetinib (μM)
180 130 100 70 55 40 35 25

# Figure 2k

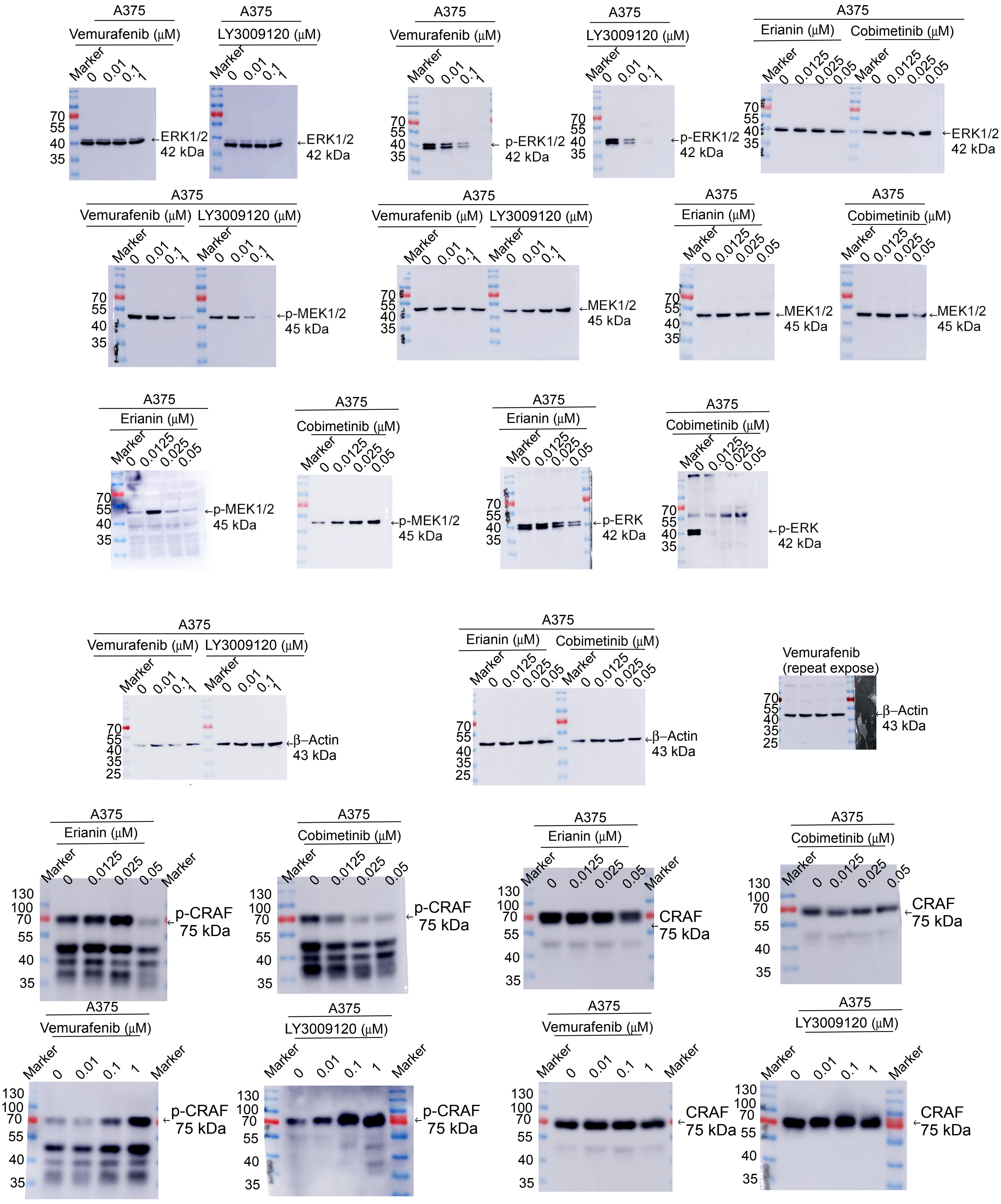
A375
Vemurafenib (μM)
Marker 0 0.01 0.1 1
70 55 40 35
ERK1/2 42 kDa
LY3009120 (μM)
p-ERK1/2 42 kDa
Erianin (μM)
Cobimetinib (μM)
Marker 0 0.0125 0.025 0.05
p-MEK1/2 45 kDa
MEK1/2 45 kDa
p-ERK 42 kDa
β–Actin 43 kDa
Vemurafenib (repeat expose)
70 55 40 35 25
p-CRAF 75 kDa
CRAF 75 kDa
130 100 70 55 40 35

# Figure 2I

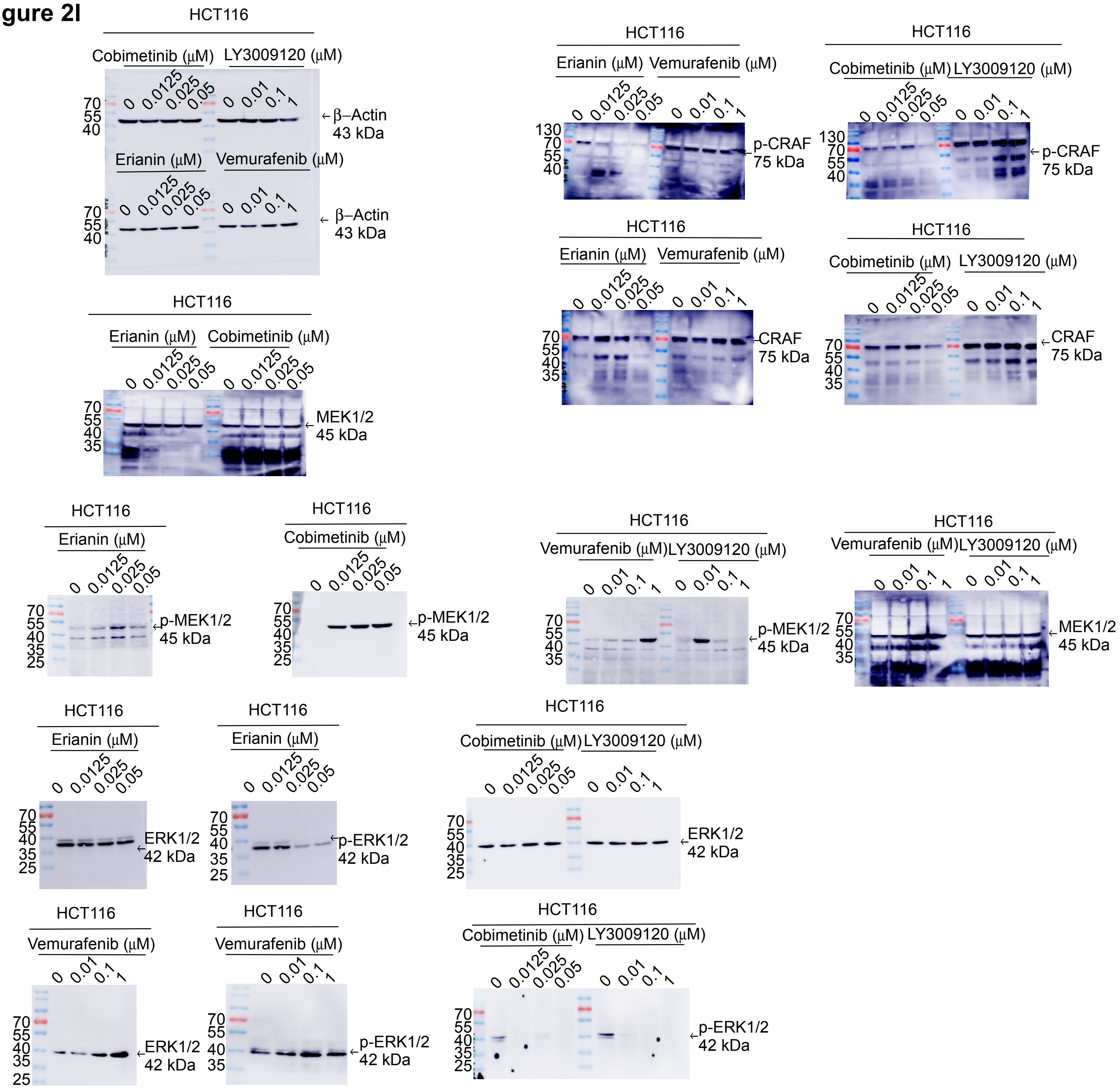
HCT116
Cobimetinib (μM) LY3009120 (μM)
0 0.0125 0.025 0.05 0 0.01 0.1 1
70 55 40
β–Actin 43 kDa
Erianin (μM) Vemurafenib (μM)
0 0.0125 0.025 0.05 0 0.01 0.1 1
70 55 40
β–Actin 43 kDa
HCT116
Erianin (μM) Vemurafenib (μM)
0 0.0125 0.025 0.05 0 0.01 0.1 1
130 70 55 40
p-CRAF 75 kDa
HCT116
Cobimetinib (μM) LY3009120 (μM)
0 0.0125 0.025 0.05 0 0.01 0.1 1
130 70 55 40
p-CRAF 75 kDa
HCT116
Erianin (μM) Vemurafenib (μM)
0 0.0125 0.025 0.05 0 0.01 0.1 1
70 55 40 35
CRAF 75 kDa
HCT116
Cobimetinib (μM) LY3009120 (μM)
0 0.0125 0.025 0.05 0 0.01 0.1 1
70 55 40 35
CRAF 75 kDa
HCT116
Erianin (μM) Cobimetinib (μM)
0 0.0125 0.025 0.05 0 0.0125 0.025 0.05
70 55 40 35
MEK1/2 45 kDa
HCT116
Erianin (μM)
0 0.0125 0.025 0.05
70 55 40 35 25
p-MEK1/2 45 kDa
HCT116
Cobimetinib (μM)
0 0.0125 0.025 0.05
70 55 40 35 25
p-MEK1/2 45 kDa
HCT116
Vemurafenib (μM) LY3009120 (μM)
0 0.01 0.1 1 0 0.01 0.1 1
70 55 40 35
p-MEK1/2 45 kDa
HCT116
Vemurafenib (μM) LY3009120 (μM)
0 0.01 0.1 1 0 0.01 0.1 1
70 55 40 35
MEK1/2 45 kDa
HCT116
Erianin (μM)
0 0.0125 0.025 0.05
70 55 40 35 25
ERK1/2 42 kDa
HCT116
Erianin (μM)
0 0.0125 0.025 0.05
70 55 40 35 25
p-ERK1/2 42 kDa
HCT116
Cobimetinib (μM) LY3009120 (μM)
0 0.0125 0.025 0.05 0 0.01 0.1 1
70 55 40 35 25
ERK1/2 42 kDa
HCT116
Vemurafenib (μM)
0 0.01 0.1 1
70 55 40 35 25
ERK1/2 42 kDa
HCT116
Vemurafenib (μM)
0 0.01 0.1 1
70 55 40 35
p-ERK1/2 42 kDa
HCT116
Cobimetinib (μM) LY3009120 (μM)
0 0.0125 0.025 0.05 0 0.01 0.1 1
70 55 40 35 25
p-ERK1/2 42 kDa

**Figure 4a**

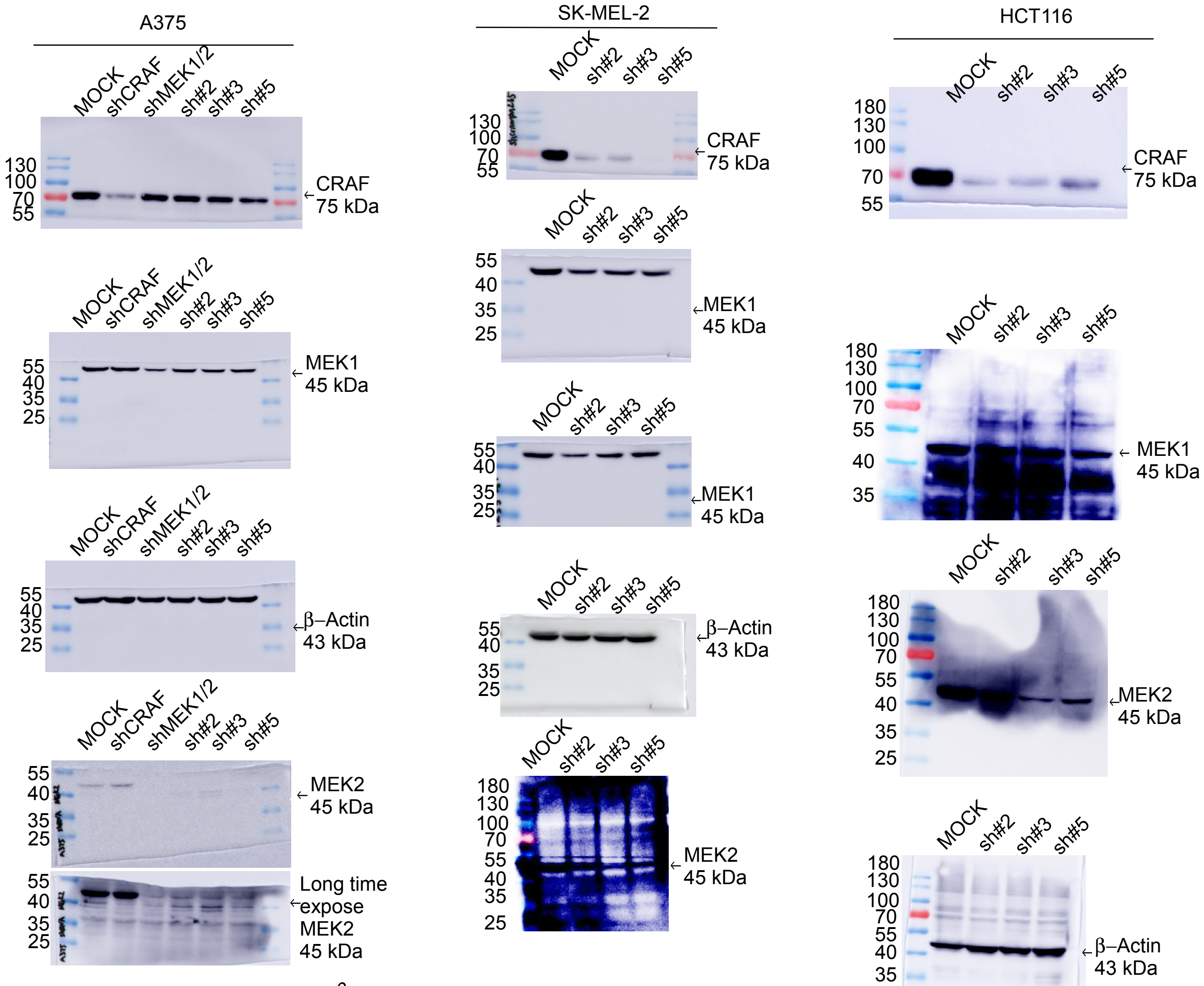


**Figure 4d**

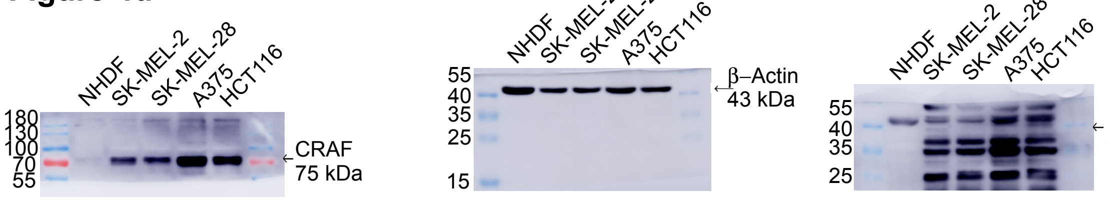


**Figure 4e**

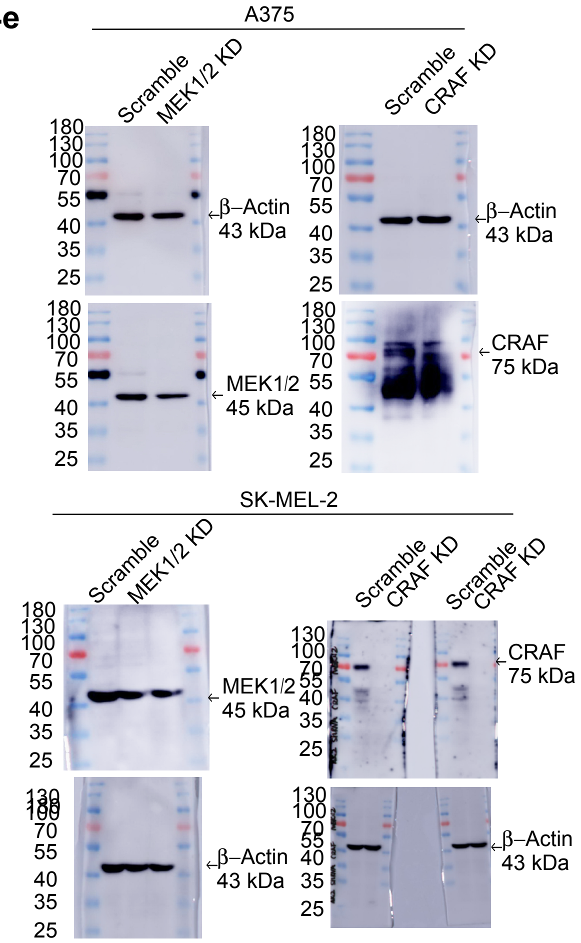


**Figure 4f**

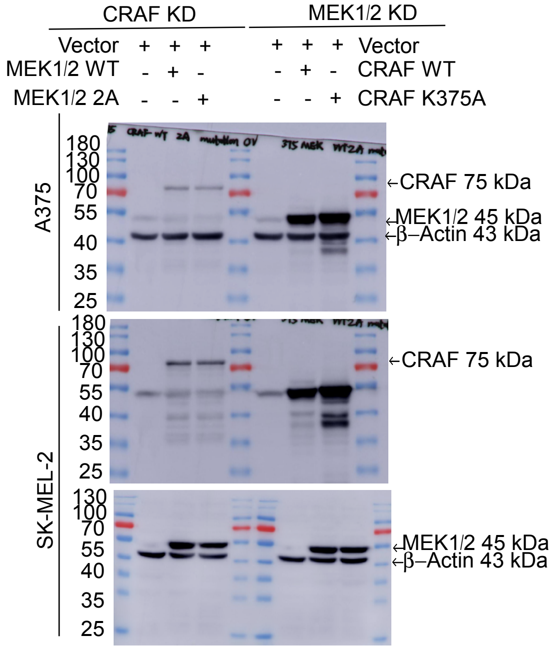

**Supplementary Figure 5c**

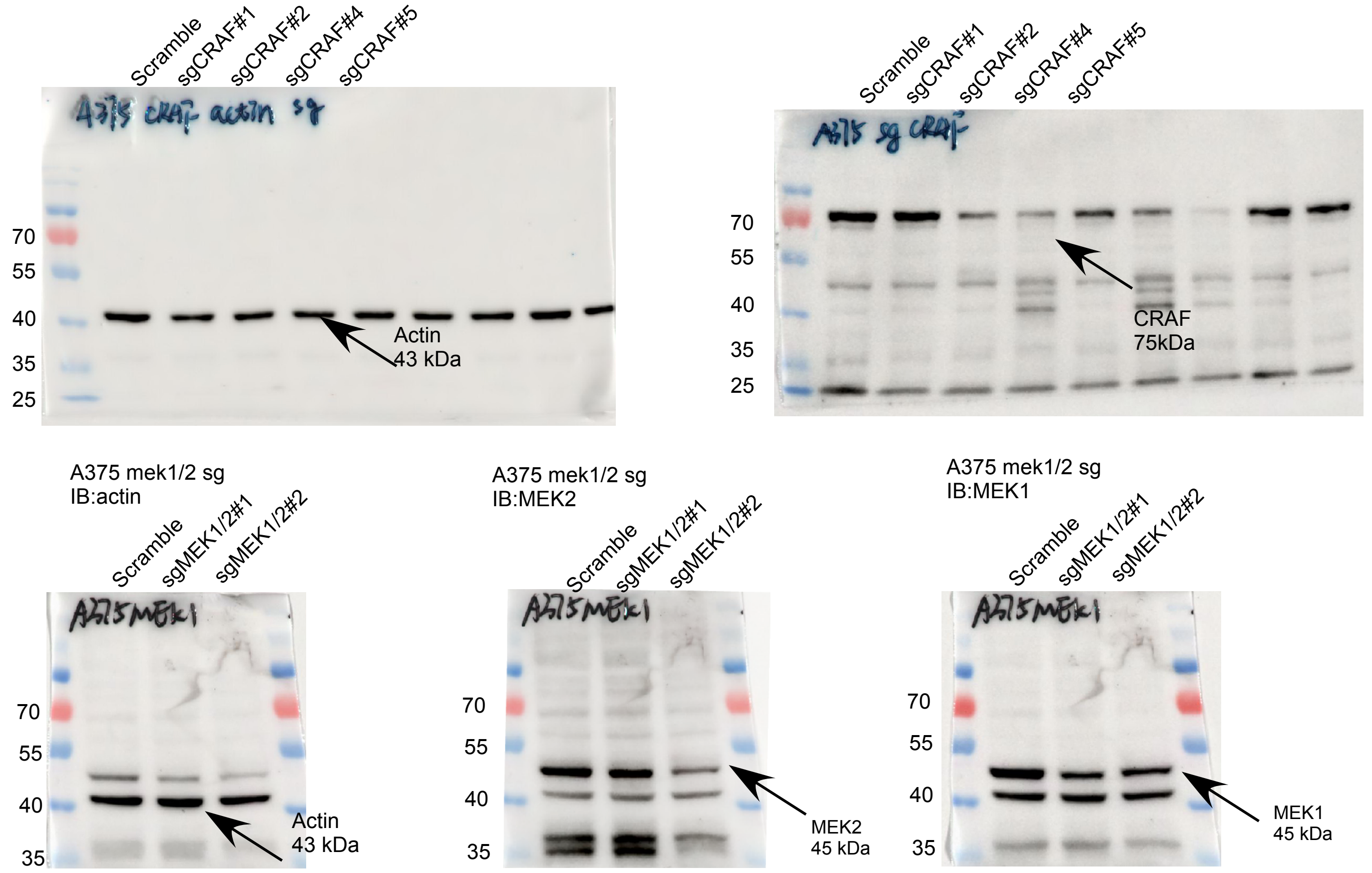


**Supplementary Figure 5d**

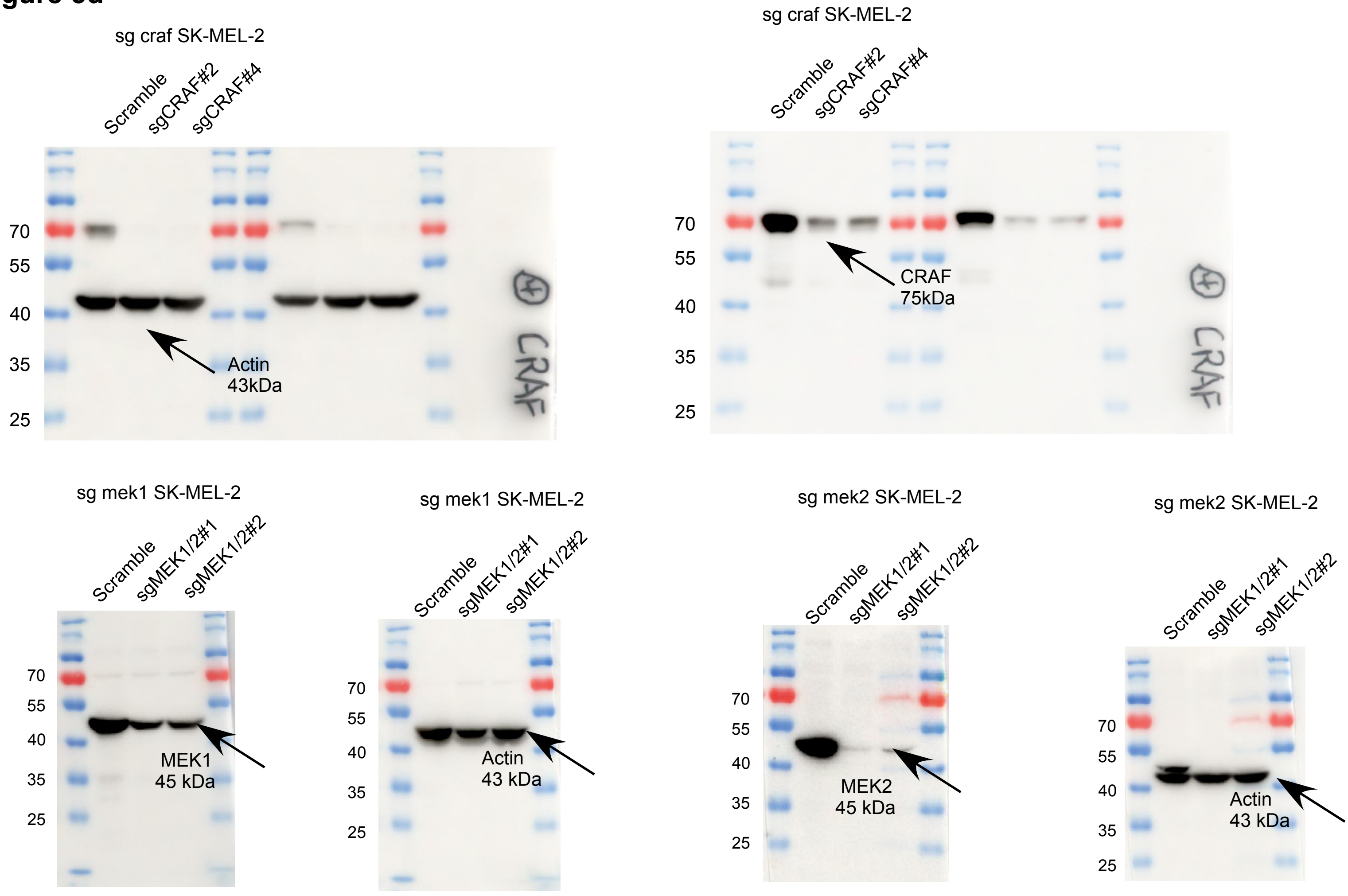

# Figure 5d-left

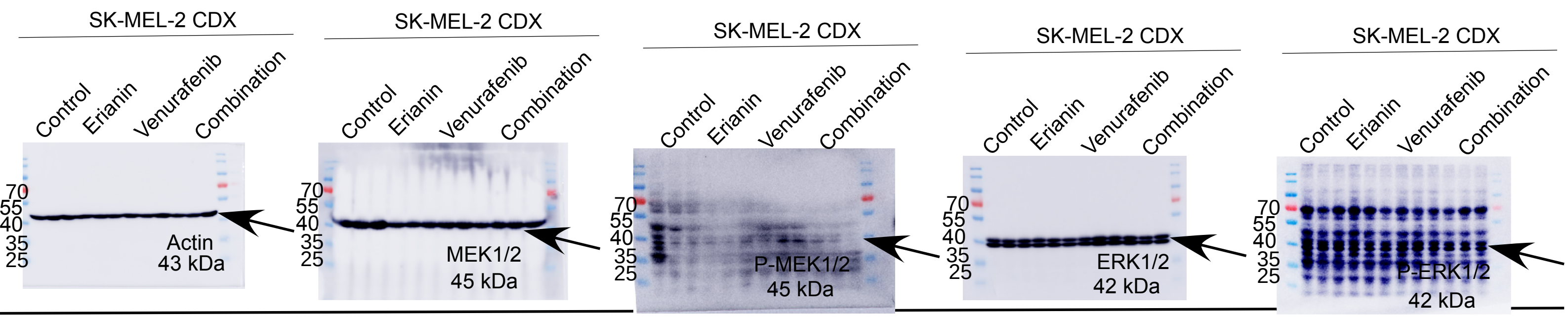


# Figure 5d-middle

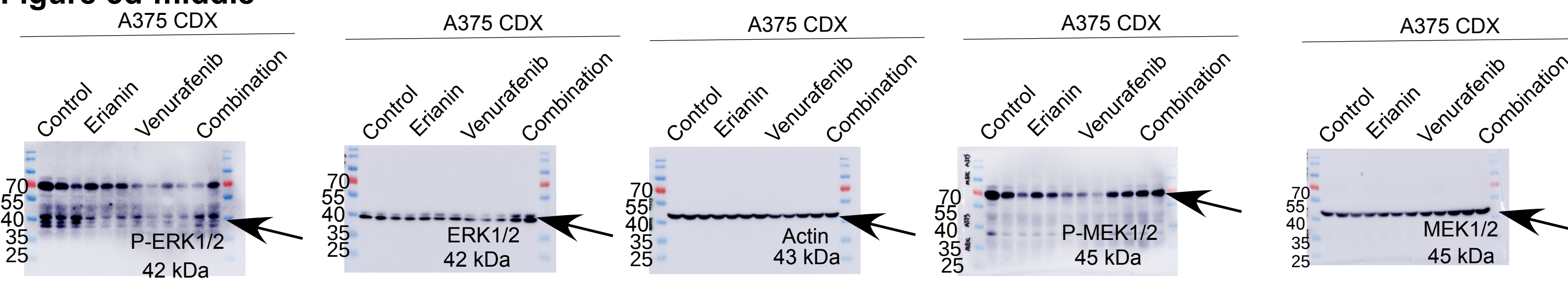


# Figure 5d-right

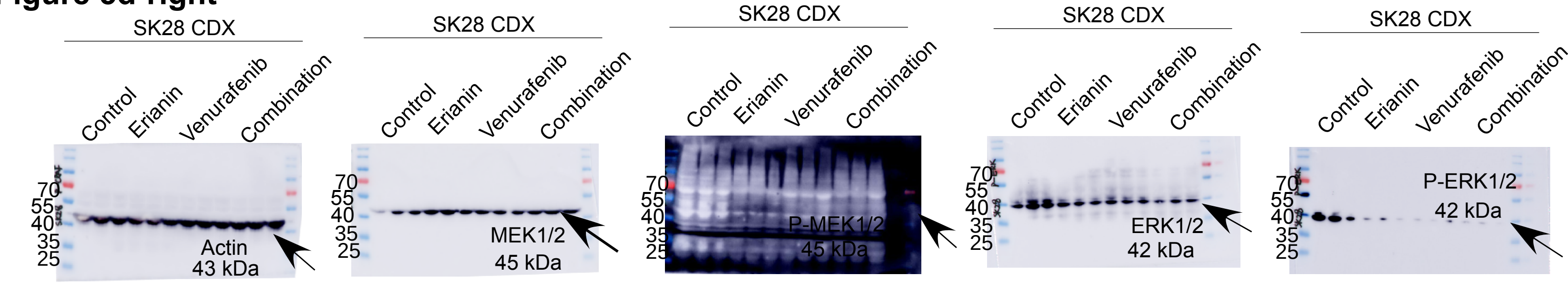


# Supplementary Fig S6b

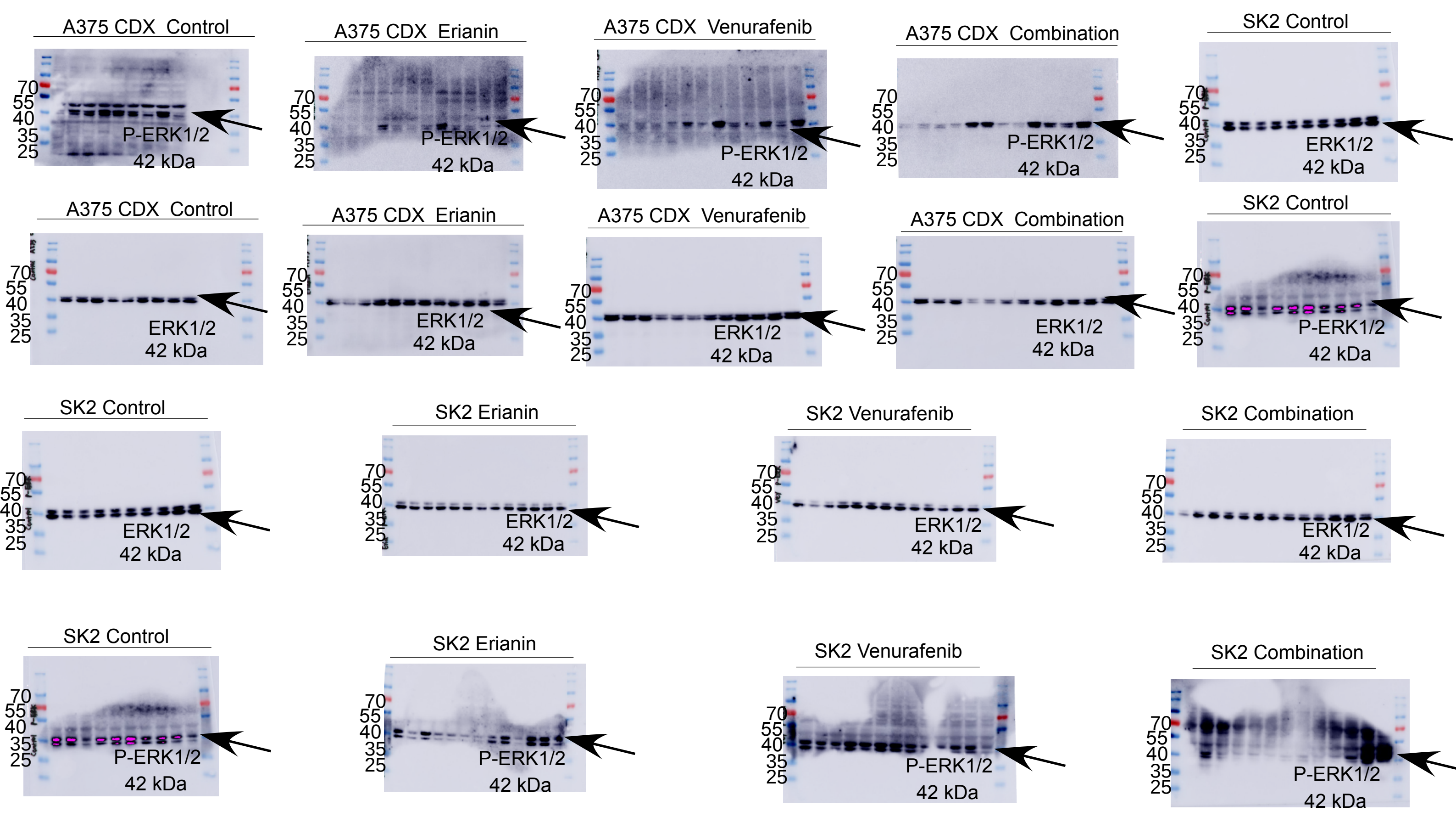

**Figure 6a**

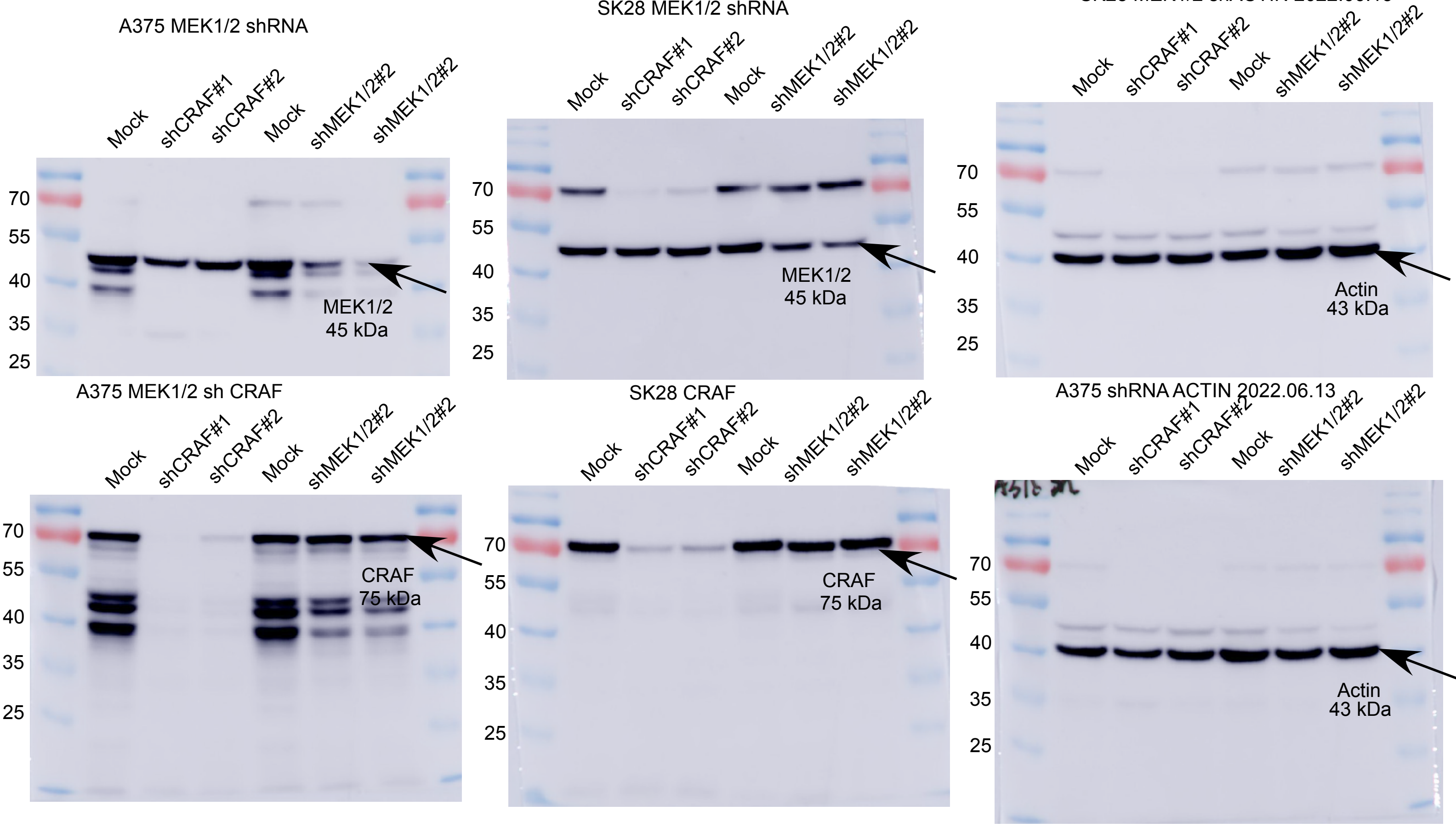


**Figure 6b**

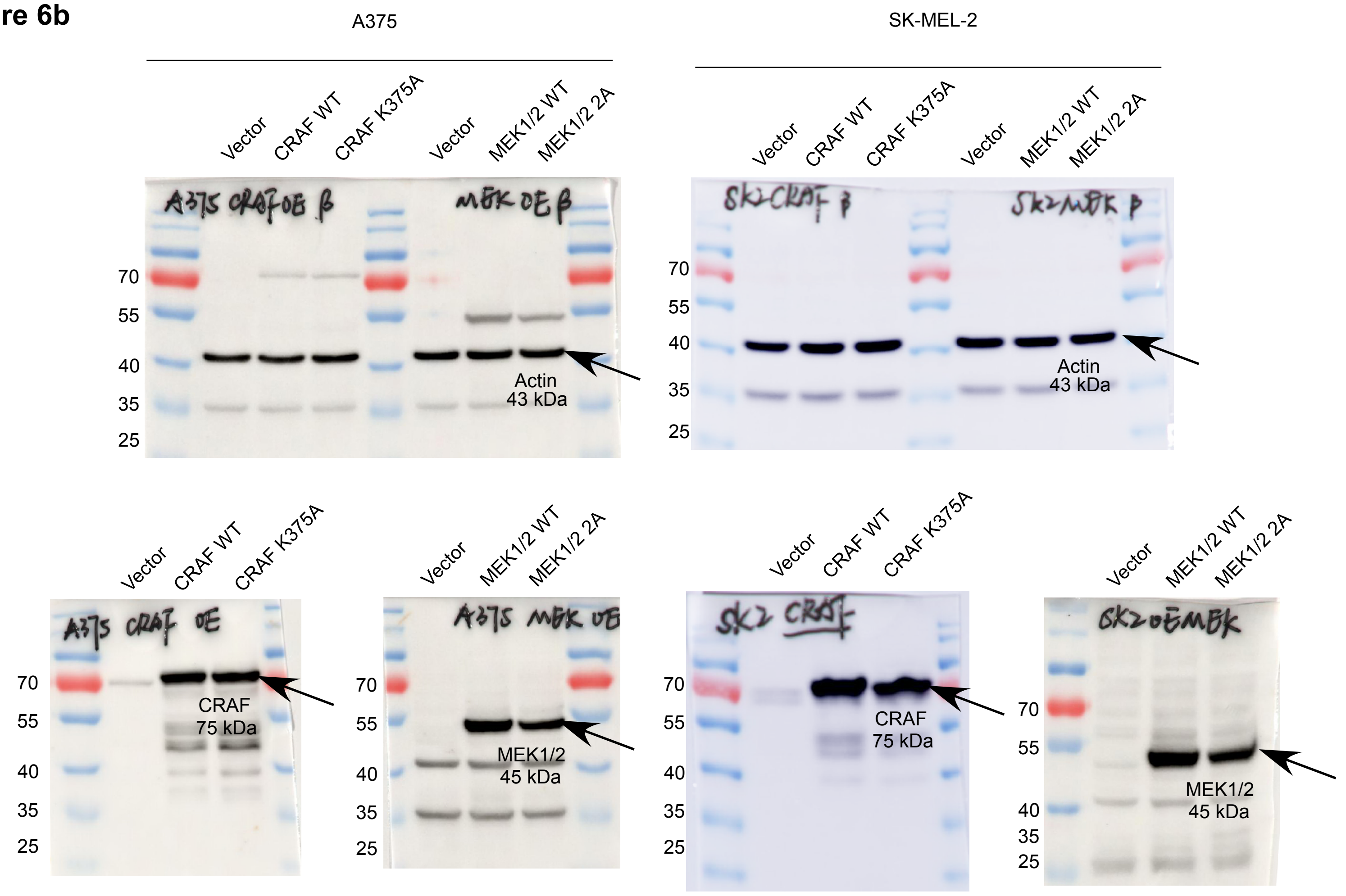

## Supplementary Fig. 7b

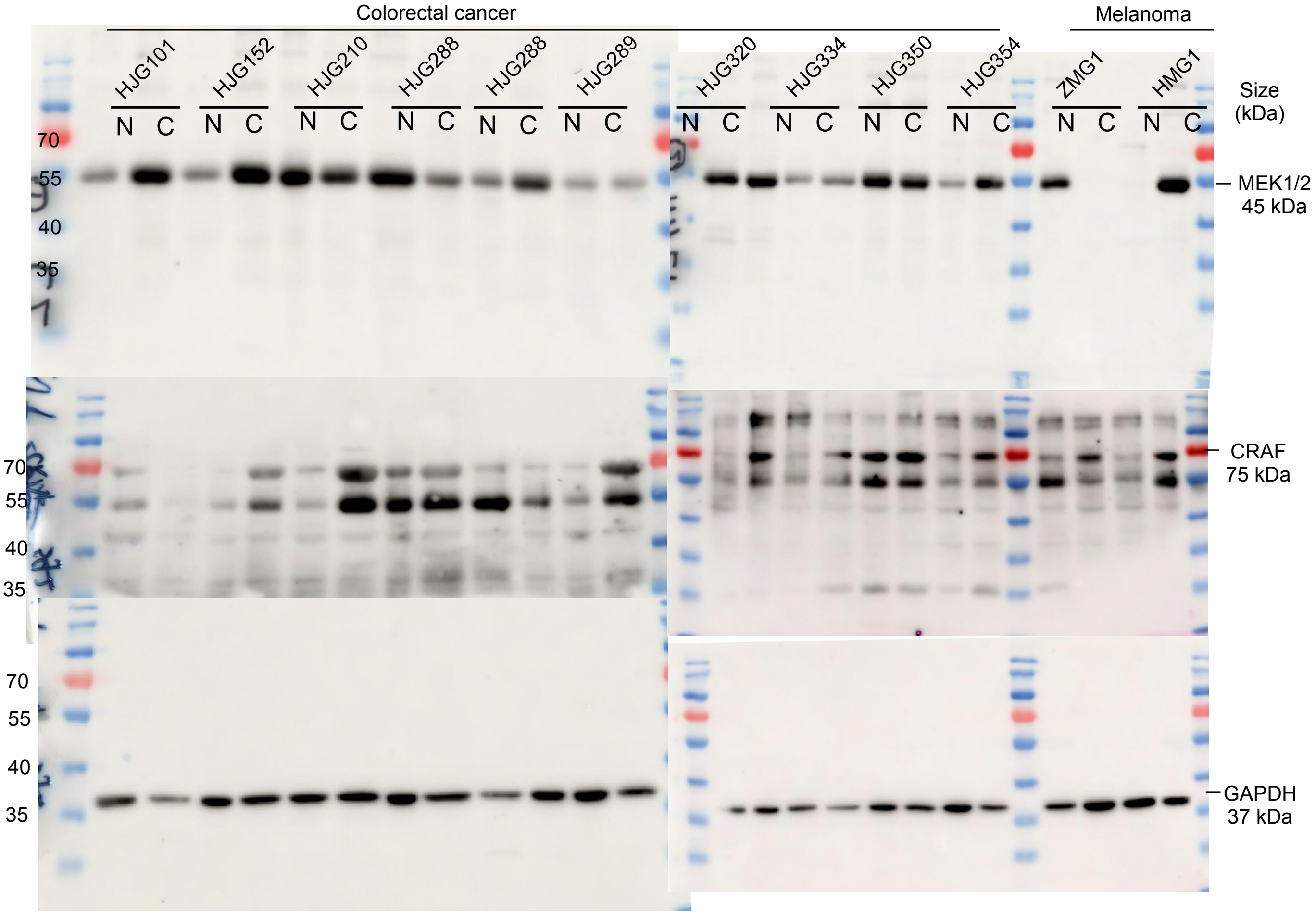

## Supplementary Figure 8c

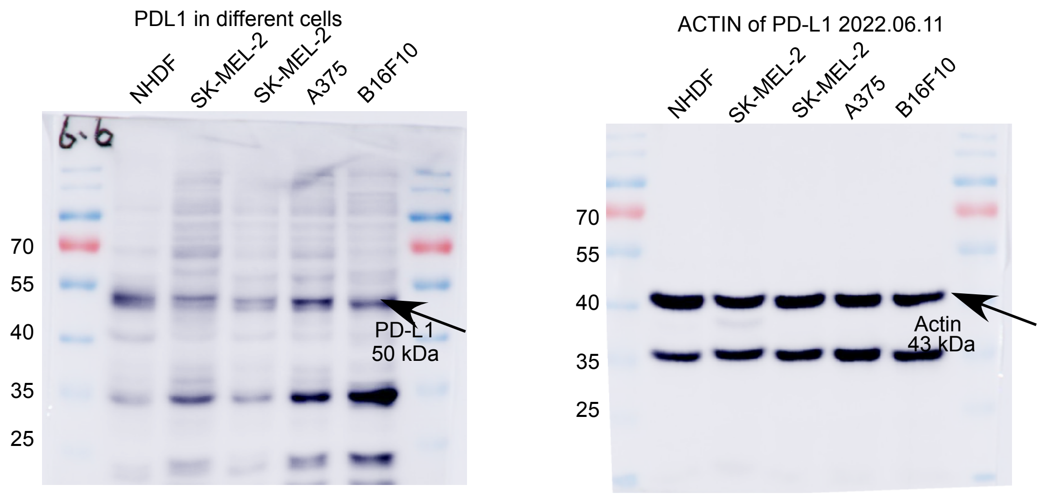


## Supplementary Figure 8d

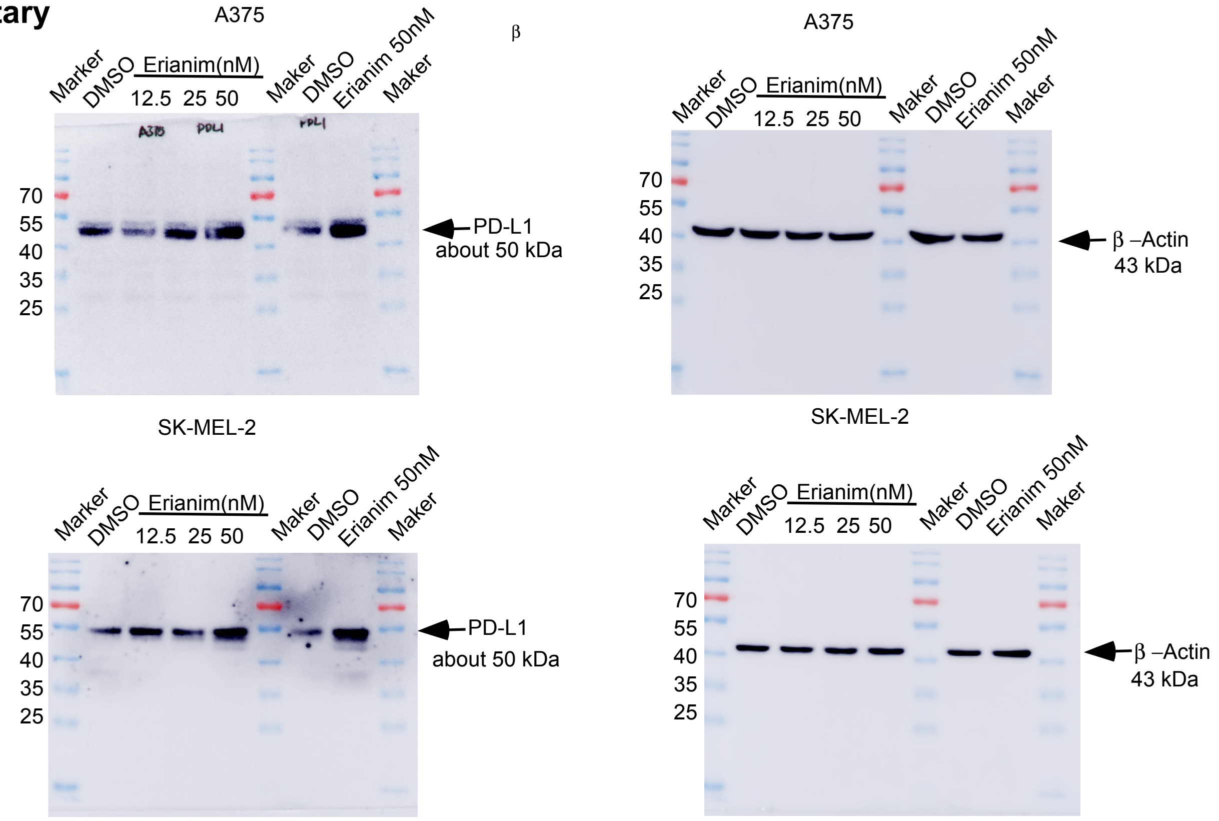


## Supplementary Figure 8f

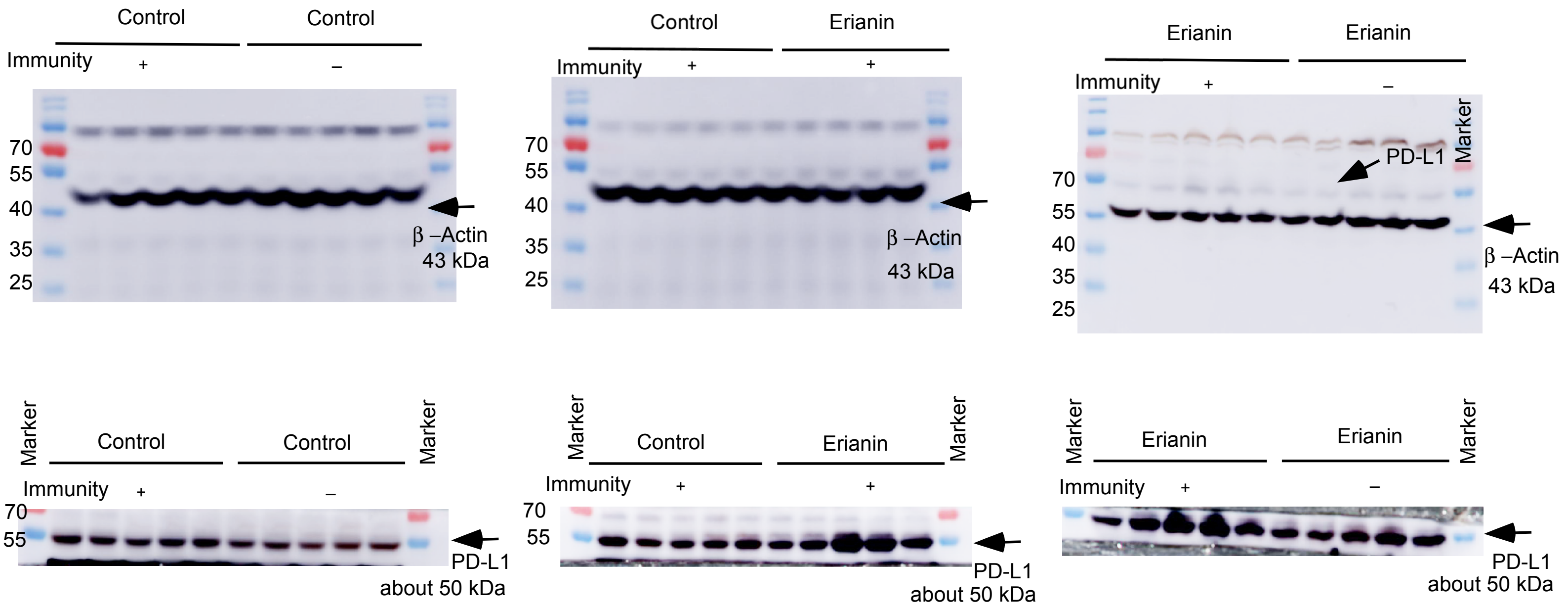

Supplement: Supplementary file 4 — Supplementary raw data [file 41392_2023_1329_MOESM4_ESM.pdf]
